# Selective recruitment of cortical neurons by electrical stimulation

**DOI:** 10.1371/journal.pcbi.1007277

**Published:** 2019-08-26

**Authors:** Maxim Komarov, Paola Malerba, Ryan Golden, Paul Nunez, Eric Halgren, Maxim Bazhenov

**Affiliations:** 1 Department of Medicine, University of California, San Diego, La Jolla, California, United States of America; 2 Neurosciences Graduate Program, University of California, San Diego, La Jolla, California, United States of America; 3 Department of Biomedical Engineering, Tulane University, New Orleans, Louisiana, United States of America; 4 Department of Neurosciences, University of California, San Diego, La Jolla, California, United States of America; Centre National de la Recherche Scientifique, FRANCE

## Abstract

Despite its critical importance in experimental and clinical neuroscience, at present there is no systematic method to predict which neural elements will be activated by a given stimulation regime. Here we develop a novel approach to model the effect of cortical stimulation on spiking probability of neurons in a volume of tissue, by applying an analytical estimate of stimulation-induced activation of different cell types across cortical layers. We utilize the morphology and properties of axonal arborization profiles obtained from publicly available anatomical reconstructions of the twelve main categories of neocortical neurons to derive the dependence of activation probability on cell type, layer and distance from the source. We then propagate this activity through the local network incorporating connectivity, synaptic and cellular properties. Our work predicts that (a) intracranial cortical stimulation induces selective activation across cell types and layers; (b) superficial anodal stimulation is more effective than cathodal at cell activation; (c) cortical surface stimulation focally activates layer I axons, and (d) there is an optimal stimulation intensity capable of eliciting cell activation lasting beyond the end of stimulation. We conclude that selective effects of cortical electrical stimulation across cell types and cortical layers are largely driven by their different axonal arborization and myelination profiles.

## Author summary

Brain stimulation is widely used to probe the neural system to learn about its properties, to normalize dysfunction (e.g., deep brain stimulation for Parkinsonian patients), or to manipulate brain activity, including enhancing memory and learning. Despite its critical importance in experimental and clinical neuroscience, at present there are no systematic methods to predict which neural elements of the brain will be activated by a given stimulation regime. To address this question, we propose a novel theoretical framework that models the effect of cortical stimulation on the spiking probability of a neuron based on its location, type and morphology. Our study predicts that short-lived superficial electrical stimulation has the ability to trigger spiking in layer IV pyramidal cells, and to evoke network activity that could persist for hundreds of milliseconds. It further predicts a much higher spiking response to anodal stimulation compared to cathodal one, as the existence of an optimal stimulation intensity, capable of inducing a maximal response in a population of cortical cells. The results of our study can be directly taken into account in planning future electrical stimulation experiments.

## Introduction

Brain stimulation is widely used to probe the properties of neural systems [1–4], to normalize dysfunction (e.g., deep brain stimulation for Parkinsonian symptoms [5–7] and epileptic patients [8], Direct-Current Stimulation for stroke patients [9]), or to manipulate brain activity, including enhancing memories and learning [10–13]. While the practice is widespread and scalable, its application is limited by the difficulty of predicting which cells (if any) are going to spike due to an input, and which specific synaptic mechanisms are going to be recruited and modulated by a given stimulation protocol [14]. In addition, the secondary effects of the directly activated neurons on other cells may be more distributed and prolonged than the direct effects themselves [15]. Furthermore, depending on the goal of stimulation, the effect of interest could be driving cells to spike or inducing sub-threshold changes. Stimulation can synchronize [16], de-synchronize [17], excite and/or suppress [18–20] neuronal activity. However, it has been difficult to infer these observations by applying the physics of current fields to cortical anatomy and physiology.

Considerable work has attempted to define the specifics of current flow when the cortex is directly stimulated [14, 21]. In particular, anatomical measures derived from brain scans, cell reconstructions, and other measures have been used to populate finite element models, leading to patient-specific suggestions regarding where current would flow for a given electrode placement [22, 23]. Such models are often “passive”, meaning the active properties of neurons (e.g., spike generation, synaptic dynamics, intrinsic currents) are not taken into account.

The effects of stimulation have been examined using electrophysiological and/or optical methods. However, the results have been ambiguous due to limitations in recording from an entire cortical volume with high temporal and spatial resolution [24, 25]. This underscores the need for careful modeling to predict plausible outcomes that can be verified separately in different layers and cell types. Furthermore, an empirically validated modeling approach could be extended easily to a variety of contemplated electrode configurations and stimulation regimes.

In this work, we develop a method that estimates the effect of extracellular electrical stimulation on the different classes of neocortical neurons. Our approach begins with calculation of the activating function [26–28], which represents the stimulation-induced effective current across neuronal membranes. Our approach is novel, in that it convolves the activating function with axonal arborizations of different cell types, obtained from public databases of over five hundred morphological reconstructions of twelve different classes of cortical neurons, while also accounting for myelination properties, morphological variability within each cell class, and the different orientations and positions of neurons. We apply this method to predict the direct activation probabilities of cortical cell types across all layers for a short (~200 *μs*) current pulse typical of clinical stimulation protocols. We then calculate the consequences of this direct activation by propagating activation through a multi-layer neocortical network model with realistic channel and synaptic elements, to predict the net effect of stimulation on network activity. Our work predicts (a) that intracranial cortical stimulation induces selective activation across cell types and layers; (b) cathodal vs anodal stimulation have distinct effects on different cell types; (c) cortical surface stimulation focally activates layer I axons, and (d) the existence of an optimal stimulation intensity capable of eliciting cell activation lasting beyond the end of stimulation.

## Results

### Distinct cell types show different profiles of activation probability

**Fig 1:**
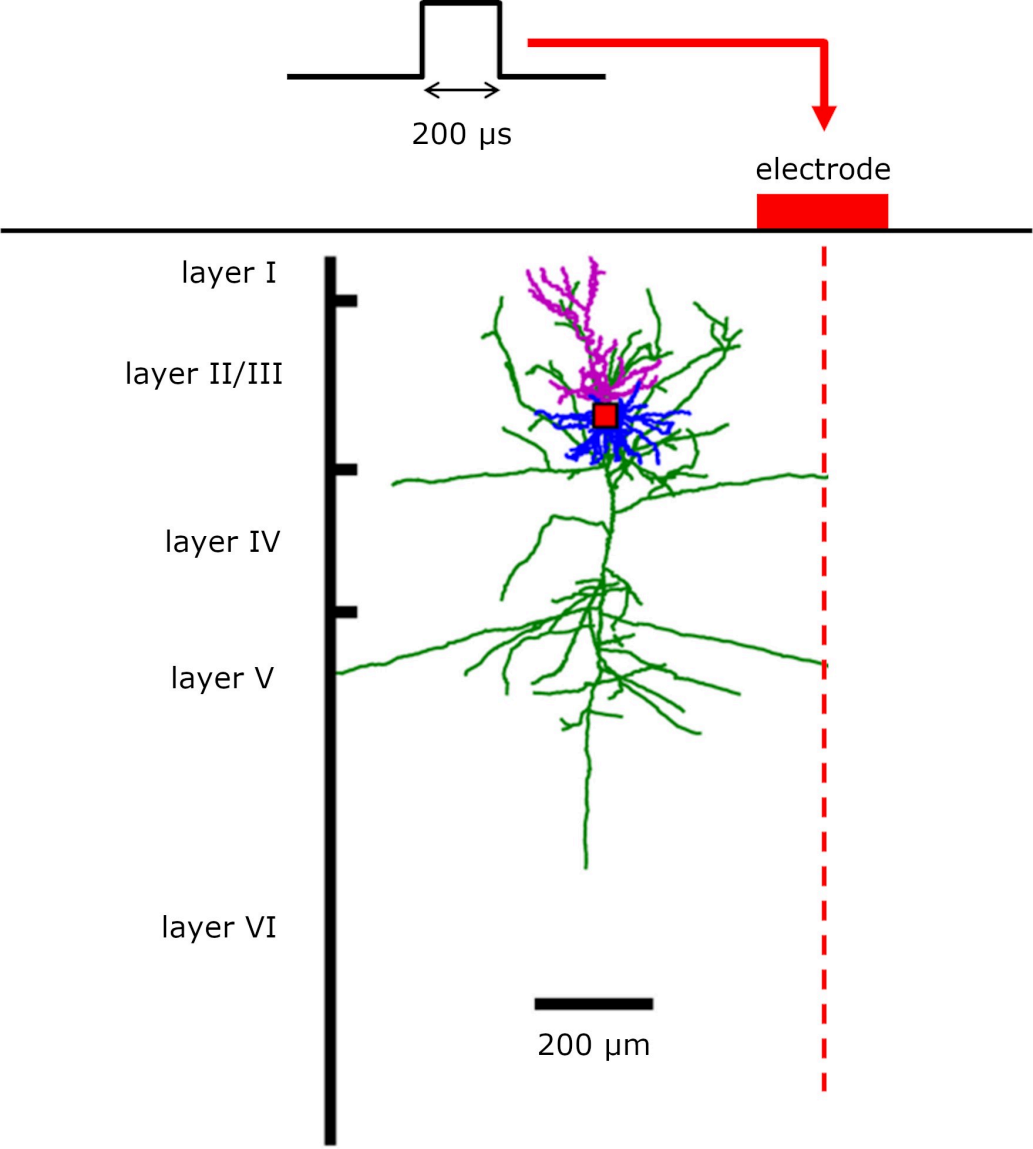
Representation of the experimental paradigm studied. An electrode on the cortical surface (squared, size 150 μm) delivers current to cells embedded in the cortical tissue (shown is one example reconstruction of pyramidal cells in layer II/III). The current pulse is monopolar, with amplitude ranging from 0 to 150 μA, and lasts 200 μs.

**Fig 2:**
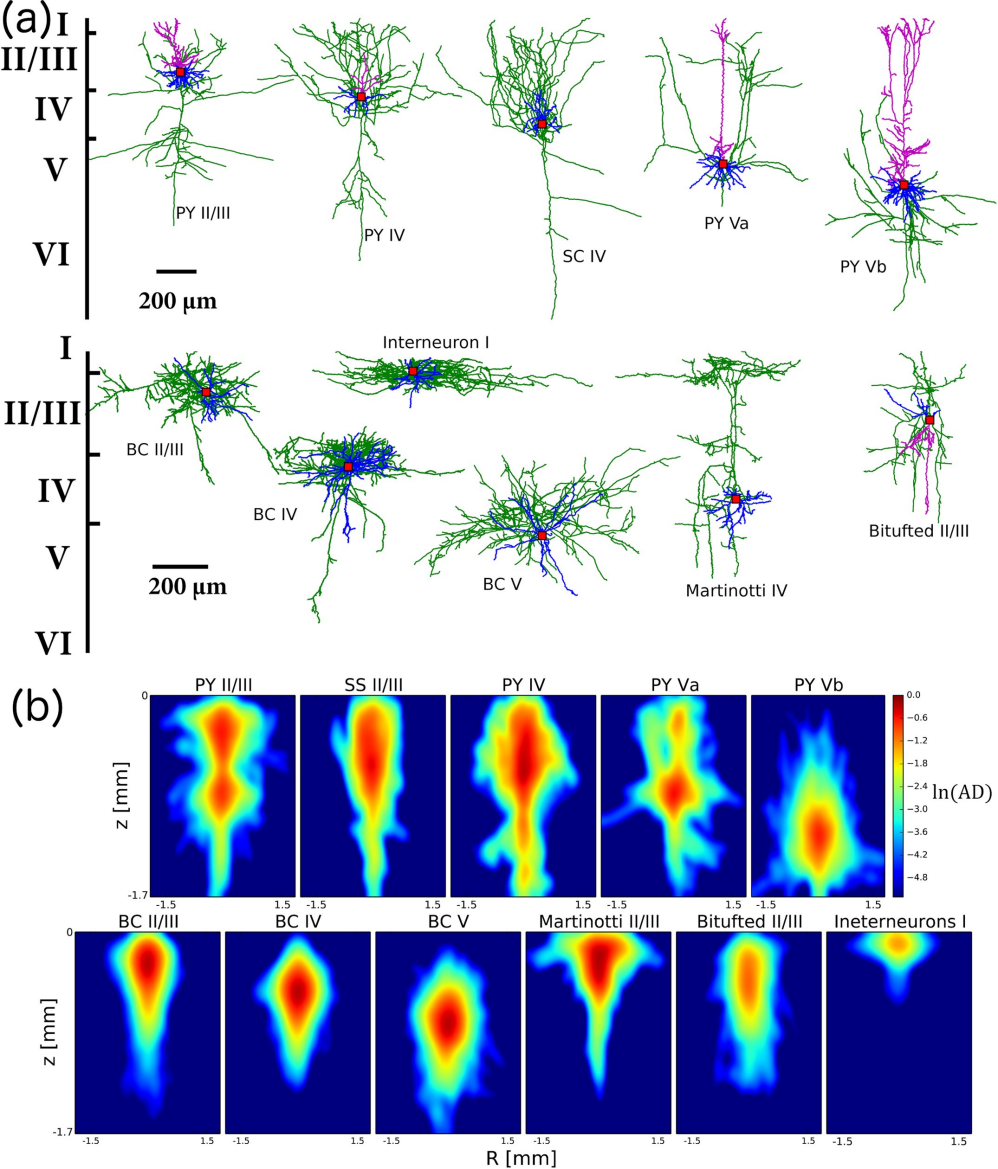
Anatomical reconstructions of the main types of cortical neurons. **(a)** Typical anatomical profiles for the main types of cortical neurons. Green denotes axon, purple–apical dendrite, blue–basal dendrite, red dot shows soma position. Top row exhibits excitatory cells (PY—pyramidal neurons, SC—spiny stellate cell). Bottom row contains inhibitory interneurons (BC—basket cell). **(b)** Averaged axonal densities formed by the neurons of each specific type. Color denotes logarithm of averaged axonal density (AD), computed over a set of available reconstructions of cortical cells. Logarithmic scale was used for better visualization of axonal arborization. This provides a general intuition on the generic shape of the axonal arborization for distinct types of cortical cells, which is crucial for the analysis.

To develop an understanding of the effects of stimulation on spiking activity of cortical cells, we focus on a specific case: an electrode on the cortical surface delivering a short-lived pulse of current (Fig 1), similar to common experimental settings [29]. Since different neuron types are distributed differently across cortical layers [30], it is legitimate to expect that their different properties and placement in the cortex would affect if and how these neurons respond to surface electrical stimulation. Hence, we resolved to study different cell types using publicly available reconstructions of cortical cells, building a collection of 561 cell anatomies, including: pyramidal cells (PYs), excitatory spiny stellate cells (SCs) from layer IV, basket cells (BCs), Martinotti cells (MCs) and bi-tufted interneurons. Our reconstruction dataset considers sub-types of PYs in layer V (slender-tufted neurons from layer Va [31, 32] and thick-tufted cells from layer Vb [33]) and BCs (large, nest and small basket cells, following a recently proposed classification [34, 35]) (Fig 2A). Criteria for inclusion of a reconstruction in our database and specific sources of each reconstructed cell are given in Materials and Methods and S1 Table. For each cell type we calculated average axonal density (Fig 2B) (see Methods: Computing the average axonal arborization for a given cell type). Averaged axonal density represents overall morphological properties of a given type of neurons (among those in S1 Table) and gives the general intuition on how the anatomy of a given cell type can influence how it is affected by electrical stimulation.

To estimate the effect of stimulation on the tissue, we assumed a squared electrode (side 150 μm) placed on the cortical surface (S1 Fig) and we calculated the electrical field potential of the current source within the tissue, based on the shape of the electrode and the total current injected into the tissue (see S1 Text).

A growing body of evidence supports the idea that electrical stimulation directly drives a response in a cell by triggering an action potential in nodes of Ranvier, or by activating the axon initial segment (orthodromic spikes) [28, 36–43]. Thus, we concentrate our analysis on estimating the effects of stimulation on cells’ axonal fibers and ignore their dendritic arborizations. We use the activating function [26–28] (computed piecewise across small portions of each axonal branch, along reconstructed axons) to estimate the probability of axonal activation. This function quantifies how much effective current locally traverses the neuronal membrane depending on its location and orientation in space (S1D Fig) upon external stimulation, and it does not account for intrinsic and synaptic currents of the neuron itself. Instead, a threshold value of the activating function can be used, such that if the activating function is larger than threshold, the small portion of axonal branch can be considered “activated” by the current field.

When estimating which threshold value we should use in our study, we aimed to introduce a realistic representation of the probability of activating a small portion of axonal branch by current, and hence we matched the experimentally measured current-distance relationship leading to direct activation of cortical cells [36, 43]. The experimental evidence was set for depth electrode stimulation, so we constructed a specific in-depth stimulation version of our estimate to obtain our experimentally driven activating function threshold (see Materials and Methods section “*Estimating the activating function threshold”* and S2 Fig). Once we estimated a threshold for each axonal segment, we considered the overall effect of stimulation on a cell by taking into account its entire axonal arbor.

**Fig 3:**
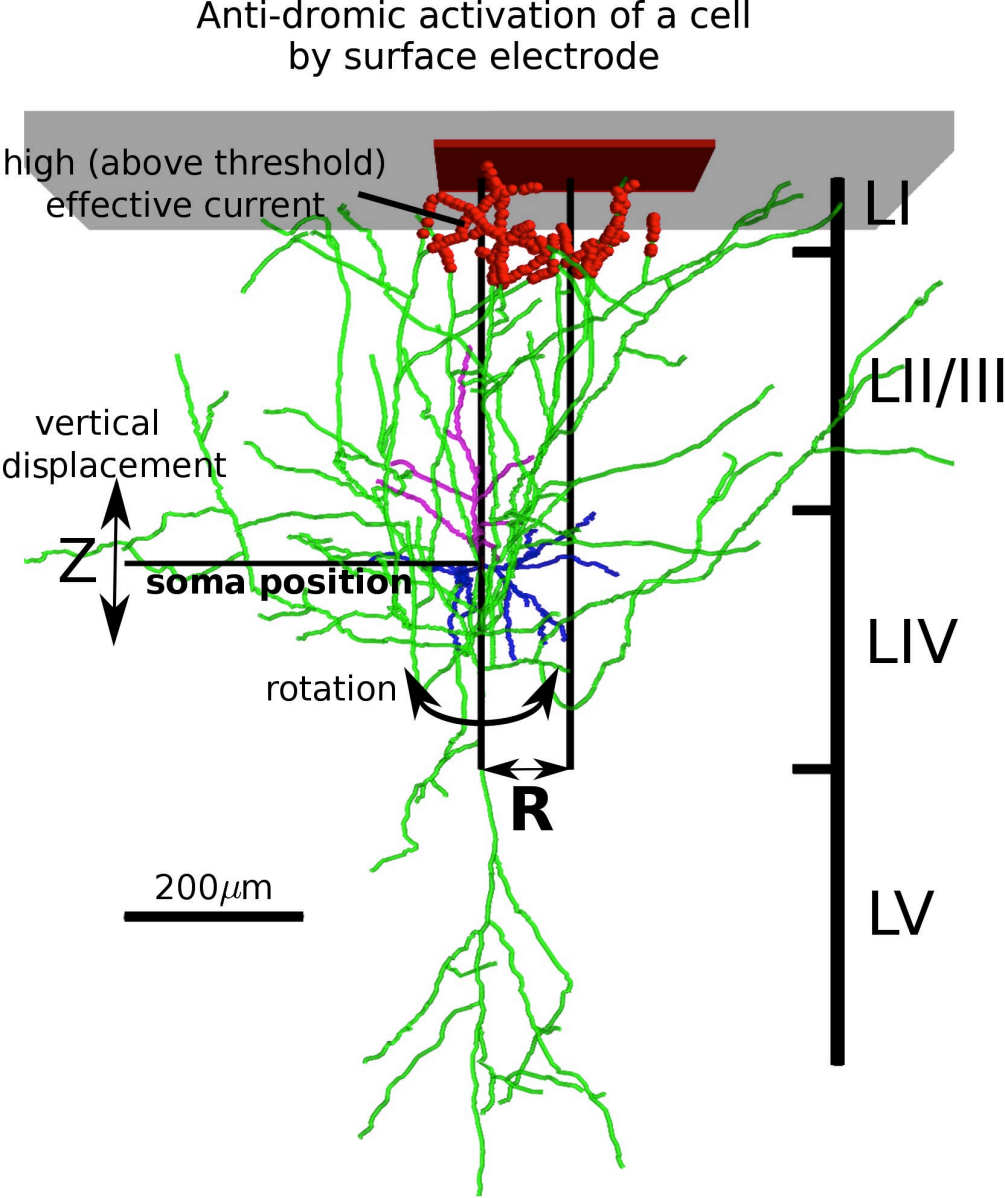
Estimation of the activation probability induced by surface stimulation. An example of typical layer IV pyramidal cell is shown. For each cell, we assigned **R**, and **Z** (depth) parameters. Activating function identifies its trigger area (red markers), where the effective current is above threshold. Action potentials can be initiated in these segments and propagate along the axonal arborization. To populate a statistical set (to find the average probability of spiking), each cell reconstruction was shuffled by rotating and shifting along the vertical axis (indicated by bold arrows), and multiple reconstructions were considered for each cell type (up to a total of 561 cells, see S1 Table and Methods: Selecting cell reconstructions within available databases).

To estimate the probability of cell activation we first computed the activating function along the entire axon arbor and, by comparing it to the threshold value, we identified which axonal segments were potentially activated (Fig 3, red markers). All together they formed a total “triggered” axonal portion, of which we knew the length (L). In case of unmyelinated fibers, the entire membrane of axon is exposed to the extracellular space and, therefore, for cell types with unmyelinated axons, we assumed a binary dependence: any L>0 (presence of trigged axon portion) produced activation, while absence of triggered portion (L = 0) meant no activation.

For the case of myelinated axons, the triggered portion could only activate the full spiking response if it included at least one node of Ranvier. Hence, we introduced a dependency of the overall probability of spike on the probability of occurrence of nodes of Ranvier in relation to the length of the triggered region. Intuitively, a larger length of the trigger area L and/or smaller internodal distance [44] along the axon lead to a higher activation probability (see Materials and Methods for details). However, it is important to note that since unmyelinated axons are less excitable their threshold of activation is much higher compared to nodes of Ranvier and axonal hillock: in our computations we used a threshold 20-fold larger for unmyelinated axons.

Since our goal was to estimate the average likelihood of activation for cells of each type, we had to account for natural variability of cell locations with respect to the current source (Fig 3). For each anatomical reconstruction of a given cell type (up to a total of 561 cells, see S1 Table and Methods: Selecting cell reconstructions within available databases), we assigned a position marking its planar distance from the center of the electrode plate (R in Fig 3), and a depth where the soma was placed within its appropriate cortical layer. To find if a cell reconstruction in that one specific placement would be activated by the electrical stimulation, we calculated its triggered portion of axonal arborization. We then rotated the cell and shifted its soma in the vertical direction (for a range of depth values that still kept the cell within its type-defining layer, see Fig 3). As a result, we obtained numerous samples for a given neuron reconstruction placed at a fixed distance from the electrode, and for each of them we evaluated if the neuron would be activated. The probability of activation for a given cell reconstruction (across all available rotations and vertical shifts) was given by the fraction of samples that were activated over the total number of samples. We repeated this procedure for each reconstructed cell belonging to a given cell type (see S1 Table), obtaining a probability of activation for each of them. We then considered the average of all these probabilities a faithful estimate of the probability of activation for a cell of a given type placed at distance R from the electrode.

**Fig 4:**
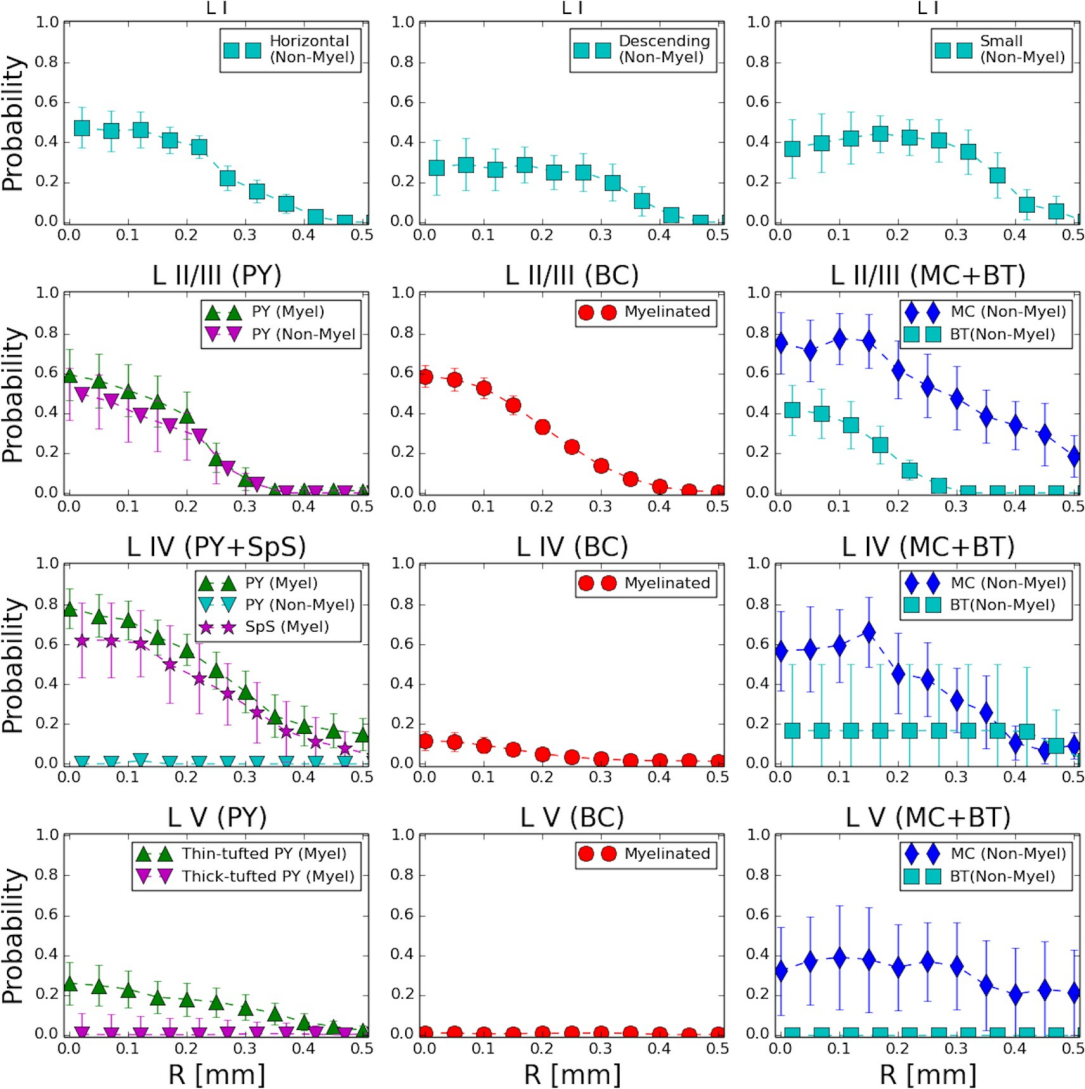
Probability of stimulation-induced activation is different across layers and cell types. The top row shows direct activation probability for 3 distinct types of layer I interneurons. Rows 2–4 (top to bottom) correspond to layers II—V. The left column contains probability for excitatory cells (pyramidal and spiny stellate), middle column contains data on soma/proximal dendrite-targeting interneurons (basket cells) and the right column contains probability for tuft/proximal dendrite-targeting interneurons (Martinotti and bitufted cells). The data represent anodal stimulation (*I* = 275 *μA*).

The method we introduced defined an activation probability function, which depended on the planar distance between a cell soma and the electrode (R in Fig 3), which could be different for different cell types. In Fig 4 we summarize the results of our probability analysis applied separately to many different cell types. Since general interpretation of experimental data [37] and our analysis (S1 Fig) suggests that anodal stimulation is most effective at depolarizing vertically oriented axonal arbors, we present the case of anodal stimulation. For clarity, we describe the different cell types in distinct sub-sections.

**Excitatory cells.** Our study predicts that pyramidal cells in most cortical layers would be only moderately activated by the superficial stimulation (Fig 4, left column, rows 2–4), with the exception of excitatory cells (PYs and SCs) in layer IV, which have a fairly high probability to spike (80% right below the electrode) in response to the current stimulation. Since layer IV excitatory cells receive input from thalamus and other subcortical structures, and locally amplify such input (by strong recurrent connectivity) before projecting it to layer II/III pyramidal cells, their higher probability of activation in response to input suggests that superficial stimulation could compensate for lacking subcortical inputs (for example following injury).

Within layer V, slender-tufted PYs (Va) showed a moderate chance of direct activation (Fig 4, bottom row, left), compared to thick-tufted PYs, which did not seem to be directly recruited by surface stimulation (Fig 4, bottom row, left). This difference is consistent with their average axonal arborizations (Fig 2): slender-tufted PYs tend to project their axons to the superficial layers [31, 32], while thick-tufted PYs axonal density is sitting away from the superficial layers. Since thick-tufted PYs are the main output of a cortical column [33], their activation effectively controls whether external electrical stimulation can influence downstream signaling to other brain regions. Hence, to activate the cortical output, external stimulation will need first to trigger enough of a local circuit response, so that the thick-tufted pyramidal cells in layer V can be recruited by the evoked neuronal activity of other cell types.

#### Basket cells

The central column of Fig 4 (rows 2–4) shows that activation of BCs is very layer-specific; in particular, layer II/III BCs are easily activated, while deeper layer BCs are much less likely to be recruited. This estimate accounts for myelination in their axonal fibers (discussed in detail below) and is consistent with the localized organization of BCs average axonal densities (Fig 2). Since BCs are the primary source of inhibition within each layer (they are the largest fraction of interneuron found in any cortical column [35]), their activation profile has the potential to shape the spiking within the cortical network. The fact that layer IV BCs are not directly recruited by input current further enables layer IV excitatory cells to trigger activity in the cortical column.

#### Other interneurons (Martinotti, Bitufted and layer I cells)

The rightmost column and top row of Fig 4 show the probability of activation for other non-parvalbumin interneuron types [35], which have unmyelinated axons and are likely to contact pyramidal cells in their distal dendrites. It is important to note that MCs from the infragranular layers (IV-VI) also target basal dendrites of the excitatory cells in layer IV [45]. Our analysis shows that MCs have high activation probability in layers II/III and V, which is consistent with their specific axonal density distribution (Fig 2). Extensive axonal arborization in layer I led to a high activation probability even though their axons are unmyelinated. Note that in our dataset, layer IV MCs show less activation than MCs in other layers, driven by the atypical shape of the axonal arbors in the reconstructions available.

Bitufted cells have moderate probability of activation only in supragranular layers. Expectedly, layer I interneurons being the closest to the current source, also exhibit high activation probability (Fig 4, top row). This predicts that a surface stimulation would recruit a fair amount of spiking in cells that are responsible for diffused and cross-layer inhibitory signaling, within and across cortical columns. It may further suggest that cortical stimulation by the surface electrode, while capable of triggering spikes in excitatory cells in deep layers, is not likely to evoke very strong excitatory events in the underlying tissue.

#### Role of myelination

The presence of myelin along an axonal fiber is considered an indication of high excitability, because the nodes of Ranvier in between myelinated segments are known to contain a high density of sodium channels [46]. Furthermore, non-uniform distribution of myelin can affect overall response to stimulation. Recently, an experimental study [47] of layer II/III pyramidal neurons revealed complex intermittent myelination patterns, where myelinated segments alternate with long unmyelinated paths. To reveal the possible impact of myelination on the activation probability of pyramidal neurons, we performed additional analysis. The leftmost column in Fig 4 compares activation probabilities for myelinated and unmyelinated pyramidal neurons. Layer II/III PYs showed no significant difference in activation probabilities due to myelination. In contrast, PYs from layer IV showed a drastic difference: with 80% activation in the presence of myelin and almost null activation probability for unmyelinated fibers.

Hence, in general, we found that cells with somas (and axonal initial segment) close enough to the superficial stimulation electrode and vertically oriented axonal arbors (like PYs in layer II/III) are likely to not see a great loss of activation probability if they lack myelination. In fact, our method shows that myelination plays a strong role in promoting cell excitability for cells which have axonal initial segments in deeper layers, further away from the current source (like PYs in layer IV), because they would rely more strongly on action potential in distal axonal fibers in the superficial layers to be generated by the input. The principle that unmyelinated fibers can be activated despite their lower excitability as long as they are placed closed enough to the current source applies also to interneurons. In fact, Bitufted cells and layer I interneurons (which are unmyelinated) show moderate activation probabilities in supragranular layers but no activation for deeper layers. Martinotti cells, although unmyelinated, do not show strong difference in their activation probability across cell layers. This is due to their specific shape, characterized by extensive axonal arborization in the upper layers.

### Type and magnitude of stimulation control cell activation probability

**Fig 5:**
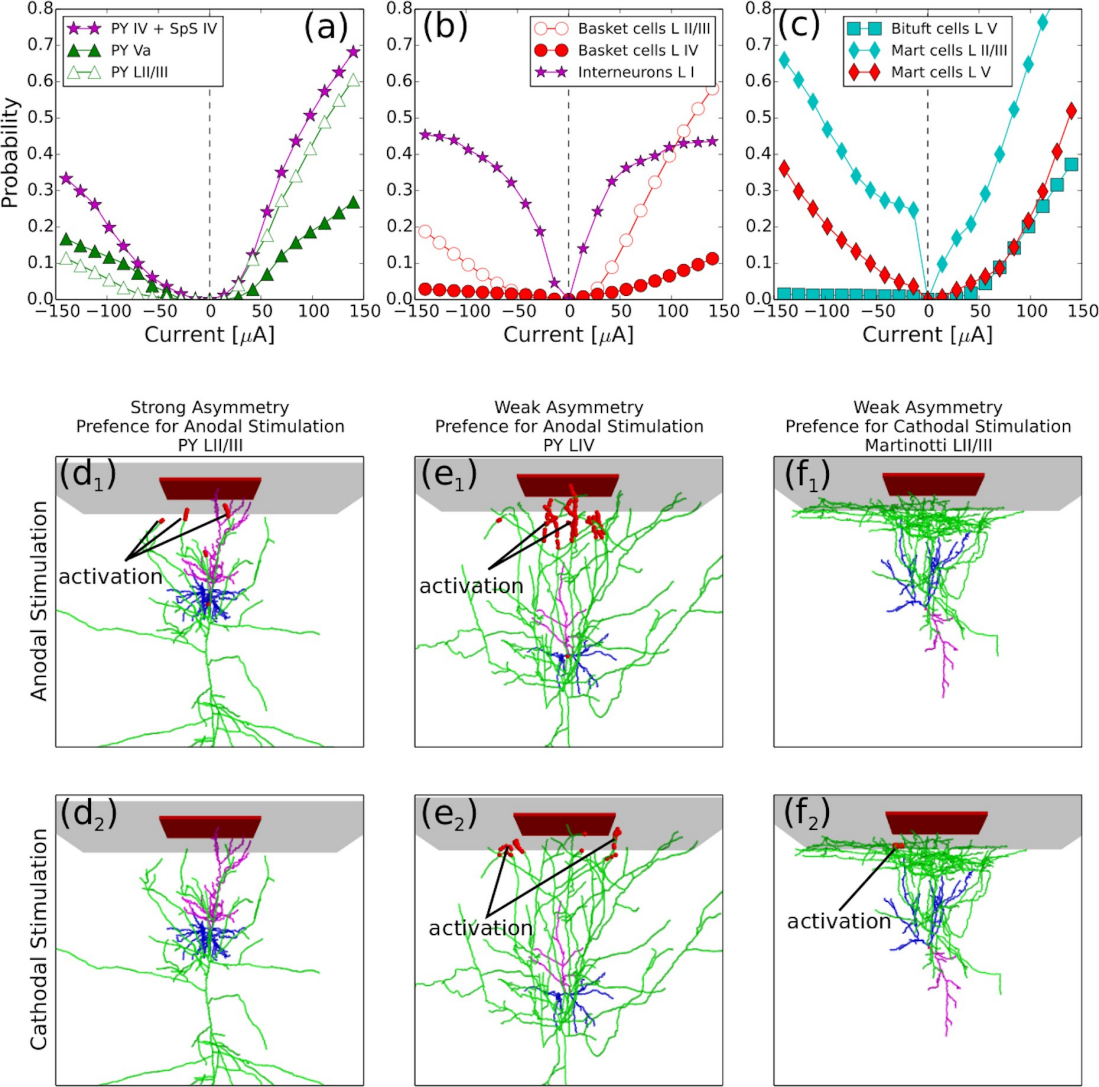
Different cell types have distinct preferences for stimulation type (anodal or cathodal). **(a-c)** Dependence of the activation probability on the net electrode current *I* for excitatory cells **(a),** basket cells **(b),** Martinotti and bi-tufted cells **(c)**. **(d**_**1,2**_**)** Anodal stimulation **(d**_**1**_**)** activates pyramidal cells LII/III more effectively than cathodal stimulation **(d**_**2**_**)**. **(e**_**1,2**_**)** Pyramidal LIV/V and spiny stellate cells have no preference for any type of stimulation. Because of the rich axonal arborization in supragranular layers, both types of stimulation provide large activation area. **(f**_**1,2**_**)** Cathodal current is more effective in activation of non-myelinated horizontal axons of Martinotti cells in supragranular layers.

In our estimates, the probability of cell activation depended directly on the portion of axonal arborization which showed activating function above threshold (length of the triggered portion). In turn, this length depended on the amount of overall stimulation current delivered by the electrode. Intuitively, a larger current magnitude induced a longer triggered portion (Fig 3), and hence a higher spiking probability. However, it is less clear how changing the stimulation polarity would affect the likelihood of cell activation (S1 Fig), since different types of stimulation have completely different effect on axonal fibers: below the electrode, anodal current depolarizes vertical fibers and hyperpolarizes horizontal fibers, while cathodal current has the opposite effect (S1 Fig). Therefore, we asked how the probabilities of cell activation (Fig 4) depended on the amplitude and polarity of the stimulating current *I*, and whether this dependence was similar across different cell types with different anatomies (Fig 2B). We applied our method to estimate activation probability when the electrode delivered different amounts of total current *I*. We estimated this probability in a volume nearby the electrode and repeated each estimate for the case of anodal and cathodal stimulation separately (Fig 5).

Fig 5A–5C shows how the overall activation probability changes as a function of the net current of stimulation for different cell types. All cells can be divided into three main categories based on their response types to a range of currents: cells which respond more strongly to anodal stimulation, cells with mild preference for responding to anodal stimulation, and cells which respond more strongly to cathodal stimulation. In fact, a cell position across cortical layers and shape of its axonal arborization defines its preference to stimulation type.

**Cells with strong preference for anodal stimulation.** Pyramidal neurons (Fig 5A), basket cells from layer II/III (Fig 5B, open circles) and bi-tufted cells (Fig 5C, squares) constitute the first class, which is characterized by strong preference for anodal stimulation. The probability dependence on the current was very asymmetric for this class of cells due to much higher probabilities for positive current. Fig 5D shows examples of the triggered portions (red markers) for a pyramidal cell from layer II/III exposed to anodal (Fig 5D upper panel, d_1_) vs cathodal (lower panel, d_2_) stimulation. As can be seen in Fig 5D_1_, anodal stimulation activated several vertically oriented branches close to the soma. In fact, in general anodal stimulation depolarizes vertical fibers (S1 Fig). In contrast, the same magnitude of cathodal stimulation was not able to produce any triggered axonal portion in this example (Fig 5D_2_), because cathodal stimulation is not effective at depolarizing vertical fibers.

#### Cells with weak preference for anodal stimulation

The second class includes cells which showed a less asymmetric relation between probability and current (Fig 5A and 5B) and hence did not show very strong preference for one type of stimulation. In this group we found spiny stellate cells, PYs from layer IV and slender-tufted PYs from layer Va. As a representative example of this case, we show in Fig 5E the triggered portion of a layer IV PY cell exposed to anodal (upper panel, e_1_) vs cathodal (lower panel, e_2_) stimulation. This cell has an extensive axonal arborization in layers I and II, containing a large number of variously oriented fibers. This arborization with no dominant directions results in a response profile which cannot differentiate between anodal and cathodal current input, because the lengths of the triggered portions created by the different types of stimulation are similar (even if specific fibers which cross the threshold are different). This is evident in Fig 5E where the red markers are different in the two panels (top and bottom) but cover a similar amount of the axonal arbor. Similar arborization profiles, with no specific dominant orientation among the axonal fibers, are typical of all cell types included in this category (Fig 2), and hence result in a similar lack of selectivity of these cells for anodal or cathodal stimulation.

#### Cells with preference for cathodal stimulation

**Fig 6:**
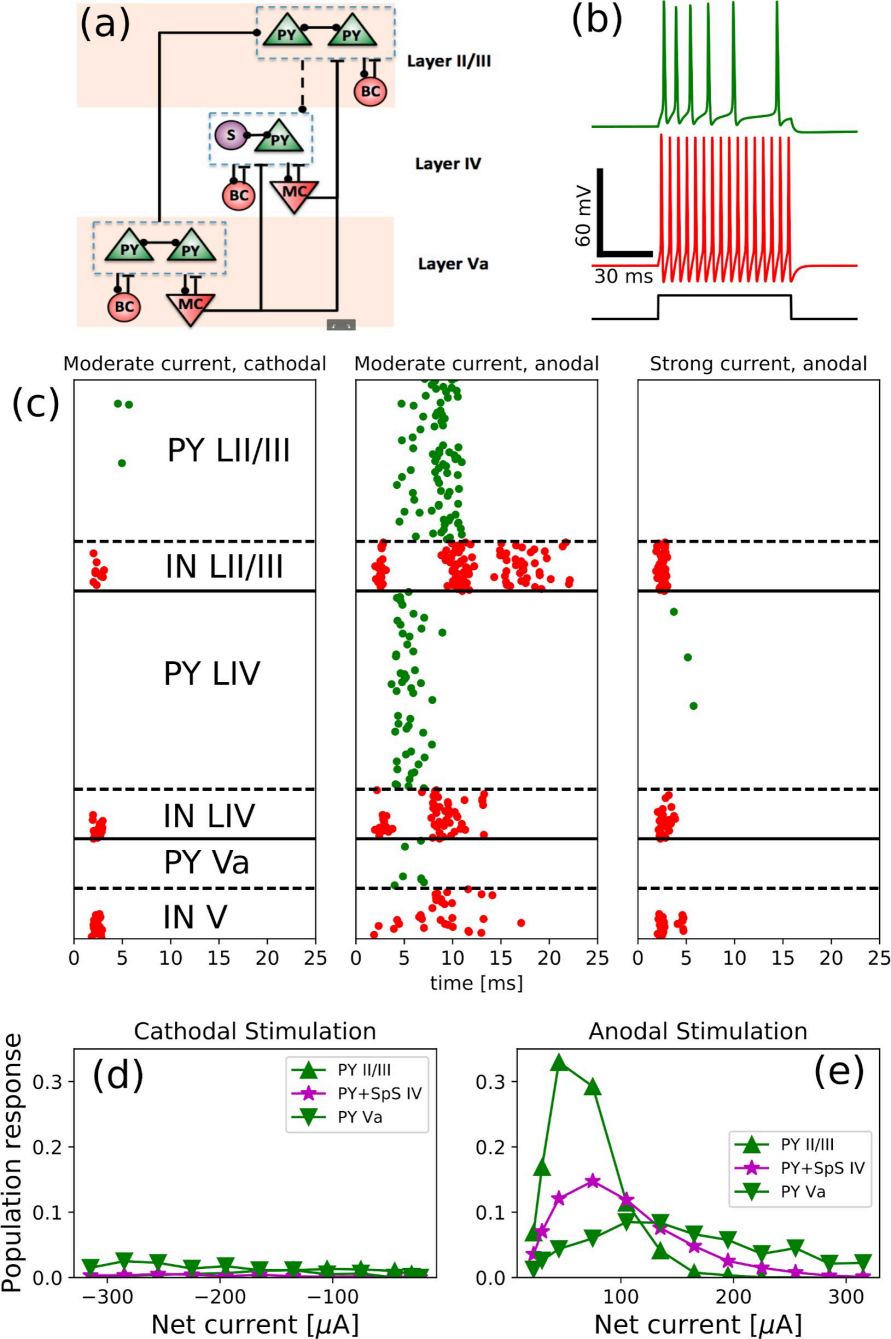
Numerical simulations predict that feedback inhibition controls response properties. (a): Schematic representation of the network model structure, which consists of 3 types of cells, located in 3 different layers (canonical circuit). PY stands for pyramidal neuron, SpS–spiny stellate cell, BC–basket cells, MC–Martinotti cells. Lines with circles denote excitatory AMPA connections (solid–strong, dashed—weak), whereas bars denote inhibitory GABA connections. (b): Two electrophysiological classes of neurons were used in our simulations: top voltage trace (green) corresponds to regular spiking neurons (used for pyramidal, spiny stellate cells and Martinotti cells) and bottom voltage trace demonstrates activity of fast spiking interneurons (used for basket cells). (c): Spike raster plots exhibit network activity for cathodal (left panel) and anodal (two right panels) stimulations. The cells were activated during first 1 ms of simulation according to activation probability (see text for details). Green dots–PY and SpS cell spikes, red dots–interneuron spikes. Left panel shows weak response to cathodal stimulation (-100 μA). Middle panel shows response to moderate anodal stimulation (75 μA), which induced a large population response. The right panel shows response to a strong positive current (300 μA), which activated a large number of basket cells in layer II/III and Martinotti cells in all layers, which prevented the activation of excitatory cells in layers II-IV. (d, e): Population responses as a function of net electrode current for layer II-IV excitatory cells.

Our analysis indicates that Martinotti cells (MCs, Fig 5V, red diamonds) are the only cell type in this group which shows a response to both stimulation types for large current magnitudes but show a strong preference for cathodal stimulation at low magnitudes (below 75 ***μ***A). The strong response at low magnitudes for cathodal current in MCs is driven by their peculiar arborization. These cells are characterized by a large number of horizontally oriented fibers in layer I (Fig 2B, Fig 6F), which are likely to get activated in the presence of cathodal stimulation (S1 Fig). Hence, even small amounts of cathodal current, not capable of reaching deep layers, still induced enough triggered portions in MCs axonal arbor (note that since MCs are unmyelinated, they do not require a large triggered portion for activation). In contrast, for stronger current magnitudes, a similar probability of MCs activation is induced by anodal or cathodal stimulation because larger currents reach deeper layers, where the overall axonal arborization of MCs also includes vertical fibers. This results in a chance for anodal stimulation to trigger activation, and hence to have effects comparable to cathodal stimulation of the same magnitude.

Interneurons from layer I do not clearly fit in one of the categories described above. In particular these cells have quite symmetric dependence of activation probability on current. This is likely because layer I interneurons are located close to the surface and can be directly activated (through axonal hillock) by anodal stimulation; conversely, their horizontally oriented axonal fibers facilitate their activation by cathodal current (similarly to MCs).

### Network simulations predict optimal currents to trigger population response and strong difference for anodal and cathodal stimulations

Our estimates of cell spiking probability driven by current input so far were all conducted for cells in isolation, ignoring the possible network effects induced by synaptic activity. To understand how considering synaptic activity could change our estimate of activation probability, we built a computational model of a cortical column, and simulated the effects of superficial current stimulation to cells in the model according to our predictions from the cells in isolation (Figs 4 and 5).

Our spiking model of cortical column included four types of neurons organized within a multi-layer connectivity structure: three distinct layers (layer II/III, layer IV and layer V) where each layer included excitatory neurons (PYs and SCs) and inhibitory interneurons (BCs and MCs). We omitted the other interneuron types because of their low density [35]. The neurons in the model were represented with point-type Hodgkin-Huxley dynamics (see Materials and Methods). Network connectivity was organized according to canonical microcircuit architecture [48–51] (Fig 6A): (a) all excitatory cells within each layer had recurrent excitatory connections, (b) PYs and SCs from layers IV and Va had strong projections to PYs from layers II/III, (c) BCs formed strong inhibitory connections to excitatory neurons within their own layers, and (d) MCs from layers IV and V made cross-layer inhibitory connections specifically targeting excitatory cells in layer IV. Two electrophysiological classes of neurons were used in the model: regular spiking neurons to represent PYs, SCs and MCs and fast spiking interneurons to represent BCs (Fig 6B). All inhibitory interneurons had lower leak current, which resulted in a higher responsiveness of these cells in comparison to excitatory neurons [52]. Parameters were chosen so that neurons would be silent if not receiving any input. (Equations, parameters and details of the rationale followed in the design of our computational model are reported in Materials and methods, *Computational model of the canonical cortical circuit: Rationale and parameters* and S1 Text). To introduce the effect of superficial current stimulation in the initial spiking probability of the neurons, we delivered a short-lived input pulse to each neuron, chosen so that all cells were activated according to the probabilities estimated from our analysis (Figs 4–6). This artificial spike-triggering input was limited to the first 200 μs of each simulation, and then turned off. This way, initial neuronal activity for each cell of specific type was defined by the spiking probability upon stimulation (derived via our estimates in Figs 4–6) but subsequently would be shaped by the synaptic interactions and intrinsic cell properties.

It is important to note that neuron models used in the network simulation were reduced models that do not explicitly represent complex morphology as discussed in the other sections. This simplification was done (1) because of the lack of information about ion channel density distribution along the cell structures for different cell types and (2) to illustrate approach that can be applied in large-scale network simulation where modeling detailed morphology is computationally impossible. Thus, instead of explicit calculations of the spiking probability, in network simulations we used the parameter *I*_*i*_^*ext*^ in each neuron to match probability estimated using activating function analysis of the isolated morphological neurons of the same type.

We tested the model for different types (anodal vs cathodal) and magnitudes of current stimulation. When current magnitudes were low, the probability of initial direct activation was low (Fig 5A–5C). In the network activity, this initial activation led to sparse spiking in few pyramidal cells and inhibitory interneurons. However, no lasting population response was evoked. Fig 6C shows representative examples of the network response to moderate and strong magnitudes of initial pulse of surface current stimulation: the three panels compare the cathodal (left) and anodal (moderate and large, center and right panels) stimulation.

In our estimates, we found that cathodal stimulation (Fig 5A–5C) triggered spiking in a small number of PY in layer II/III but activated a large number of MCs and layer I interneurons in comparison to anodal stimulation. Hence, we hypothesize that cathodal stimulation could recruit strong inhibition in the full network model and produce weaker overall network response. When tested in simulations, this hypothesis held true. In fact, cathodal stimulation evoked spikes in a large number of MCs in all layers, including layer IV and V (red dots) where they constitute a large fraction of all interneurons [35]. Note that MCs from infragranular layers specifically target excitatory cells in layer IV [45]. Hence, the effective recruitment of inhibition and relatively small activation probability of layer II/III PYs resulted in a weak and sparse response of excitatory cells in our model column (left panel in Fig 6C).

The middle panel of Fig 6C shows that moderate anodal stimulation could trigger network activity across the layers for a considerable period after stimulus offset. In fact, stimulation triggered abundant initial spiking in mutually excitatory PYs and SCs in layer IV, and some spikes in PYs in layer II/III. In turn, the connectivity within layer IV created reverberating excitatory activity that promoted a strong local network response, which projected onto layer II/III PYs, eliciting a strong population response within layer II/III. While activity of PYs in layers IV and II/III did outlast the current input, it also recruited feedback inhibition from BCs and MCs, leading to termination of the response activity. Our simulations show that anodal currents of moderate magnitudes can induce a functional response beyond the stimulation duration and location. In fact, firing of layer II/III PYs had the potential to reach other columns and deep layers PYs, which could then transmit to other areas the activity elicited in the network by the brief surface stimulus.

In case of strong anodal stimulation, our estimates showed a high probability of activation for most cell types (Fig 5A–5C). In the full network, our simulations showed a response dominated by inhibitory activity (Fig 6C, rightmost panel). In fact, current stimulation initially activated both interneurons and excitatory cells, but the larger input resistance and higher responsiveness of interneurons compared to PYs and SCs resulted in the activation of these inhibitory neurons before the spikes in excitatory cells. Because of the large number of interneuron spikes evoked, synaptic inhibition on PYs and SCs could overcome the excitatory drive due to stimulation, and effectively stopped them from firing. Because of the lack of directly recruited inhibition in layer Va, slender-tufted PYs were activated by stimulation, but their relatively low density meant that they could only deliver a small amount of excitation to the highly inhibited upper layers.

While Fig 6C shows few characteristic examples, panels Fig 6D and 6E summarize the findings across simulations with many current magnitudes. These plots show the average population response (computed as a number of spikes divided by the total population number) for excitatory cells as a function of applied stimulation current *I* (cathodal in panel 6d and anodal in panel 6e). For low current, probability of direct activation was low in both anodal and cathodal stimulations, whereas for large current, inhibitory activity suppressed spiking in PYs and SCs. Importantly, for excitatory cells and anodal stimulation there was an optimal range of stimulation current magnitudes where population response was highest (consistent with our description of network dynamics in Fig 6C, middle panel). In fact, the input evoked moderate amount of excitatory spiking amplified by recurrent excitation, which led to the high overall response in the cortical column.

## Discussion

In this study we estimated the probability of activation for different cell types and in different cortical layers when exposed to external electrical stimulation. We present the case of a finite size electrode placed on the cortical surface. The approach consists of 4 main steps: first, estimate the electric field potential in the tissue; second, define the ‘axonal-electrical receptive field’ based on reconstructions of different cell types across layers; third, estimate cells’ spiking probability based on the activating function in axonal elements; finally, predict the network response to stimulation in a model of a cortical column based on the spiking probability estimates. Our study predicts that short-lived superficial stimulation with a single electrode source has ability to trigger spiking in layer IV pyramidal cells, and to evoke network activity that could persist for hundreds of milliseconds. It further predicts a much higher spiking response to anodal stimulation compared to cathodal one, as well as existence of the optimal stimulation intensity, capable to induce a maximal response in a population of cortical cells.

### Relevance of our findings to existing stimulation protocols

Recent advances in techniques of multisite cortical stimulation [53], aimed to restoring damaged brain operations like movement, sensation, perception, memory storage and retrieval, underscore the need for better understanding the effects of such stimulation on individual neurons and synaptic connections. Our study predicts that local electrical stimulation may elicit activity under a superficial electrode sufficient to enable signal transmission without trumping the physiology of the network. Indeed, in our study, stimulation within an intermediate range of positive input currents triggered network response that survived the end of the stimulus, and thus can potentially be similar to the physiological processing. The last is important if the stimulation targets to induce physiologically relevant persistent changes in brain tissue. For example, inducing synaptic plasticity is necessary for stimulation to be successful as an intervention to restore memories [54, 55], promote the recovery of a cortical area lost functions [56, 57], or repress hyper-excitability of a portion of tissue [58]. However, blanket plasticity evoked by the cell spiking that is directly triggered by external current is not likely to match existing synaptic patterns and will not have any meaningful constructive impact on a system of careful counterbalances like the brain. In contrast, stimulating in the range of currents that elicit network-driven activity that continues when the stimulus is removed provides a better chance for the stimulation to be changing only a selected and physiologically meaningful subset of synapses. In other words, there is a range of current values in which stimulation can be used as a sophisticated and detailed intervention rather than a blunt hammer, and such range can be found using approaches combining theoretical estimates of the current density and reconstructed anatomy.

Our work focuses on the effects of a single brief isolated pulse, in contrast to repetitive trains or longer-lasting modulation, which are typically used in the clinical setting (such as in cortical stimulation mapping, neurorehabilitation with tDCS, etc.). Also, clinical stimulation is often bipolar (between two electrodes) and biphasic, with anodal and cathodal current balanced in order to prevent irreversible Faradic currents [59, 60]. Furthermore, our charge density is ~0.06μC/cm2, far below levels commonly used in clinical stimulation. Of course, activation thresholds are probably higher in humans due to thicker pia and cortex, but detailed neuronal reconstructions are not available to model the effects of these anatomical species differences. Thus, our results, as presented, cannot be applied directly to the usual clinical context, and future studies are necessary to connect our findings to the clinical realm. Specifically, in the case of weak and long-time scale current waveforms (like tDCS) the stimulation is capable of modulating cell properties, and our method would need to incorporate estimates of the dendritic dynamics in the presence of extracellular electric fields (for example using the equivalent cylinder models [61, 62]. Also, trains of stimulation pulses (such as in cortical mapping) will have effects beyond the simple summation of the effects of single pulses, due to intrinsic cell dynamics and synaptic interactions, and both dendritic and axonal dynamics will need to be included in the spiking probability estimates (using for example multi-compartmental models [63]. However, as demonstrated experimentally [43], the threshold for evoking action potentials by stimulation of the type we consider is by far the lowest at the nodes of Ranvier, and next lowest at the axon hillock. While transmembrane currents can be conducted intracellularly from the dendritic tree to the axon hillock, they are greatly delayed and attenuated, and their effects are minor compared to the direct stimulation of these elements.

Our results suggest that superficial anodal stimulation is more effective than cathodal at cell activation. Clinically, with respect to cathodal vs anodal stimulation effects, the evidence is somewhat mixed. Previous work by [64], among others found that surface-anodal stimulation had lower threshold for activation of corticofugal fibers in the Baboon’s motor cortex. In Pollen’s classic studies [65] recording units in cat visual cortex in response to stimulation of the overlying surface, he noted that different cells had lower threshold to either cathodal or anodal currents. A possible explanation can be found in the modeling study [66] which found that although surface-anodal current would preferentially activate vertically oriented elements directly beneath the electrode (as in our study), elements in the sulci would activated by cathodal current, and these effects interacted with the precise location of the electrodes with respect to gyral crowns and whether bipolar stimulation was used.

### Limitations of the approach

Experimental studies over past decades found that electrical microstimulation directly activates axon initial segments and nodes of Ranvier [28, 38–42], which are the most excitable elements due to the high concentration of sodium channels [67]. Thus, the estimation of direct activation of cortical cells in our study was based on calculation of the activating function along the axonal segments. This approach takes into account orientation and thickness of axons, and also considers their myelination properties. A previous study reported that this approach may overestimate the activation of threshold currents at distances >250 *μm* [68], however myelinated fibers were excluded from the analyses, so it is unclear if and how this consideration would apply to the current study.

An alternative to the activating function approach could have been to use the mirror estimate, which has been shown to be a better predictor of the steady-state intracellular potential given an extracellular potential field under most commonly used experimental conditions and preparations [69]. However, given the short duration (20 μs) of the electrical pulse we studied, both approaches have been found to give comparable estimates [69]. Moreover, we were interested in the transient effects of electrical stimulation, rather than the resulting steady-state, and it has been shown that the activating function is better suited to estimating these types of effects than the mirror estimate [69]. We chose to concern ourselves with the transient, initial response to electrical stimulation due to both the short duration of our simulated electrical pulse, and the fact that we used passive cable theory to model our dendritic and axonal arbors, and thus could not account for much of the dynamic phenomena which would result as the steady-state was approached. In future research, if longer pulse durations are studied and the steady-state response is the phenomenon under consideration, the mirror estimate would be the more appropriate approach.

There are other methods that could be applied to directly estimate the activation threshold based on detailed computational models of the dynamics of each neuron type. These methods must incorporate linear and non-linear conductances using to specifically account for lateral spread of currents across neuronal compartments. This level of detail is not achieved in the electrostatic models that we used in this new study, which are focused on establishing how neuronal morphologies influence the differential level of response of different cell types to the same incoming stimulus. One advantage of our approach, however, is that it can be applied to a broad range of different types of neurons where neuronal morphology has been characterized (i.e., almost all main classes of vertebrate neurons), versus the very limited range of neuron types where there are sufficient data on the distribution of ion channels across neuronal membranes to model the relevant conductances.

We defined a threshold for the effective stimulation current in an axonal segment (given by the activating function) by direct comparison to the experimental data: in turn, this threshold enabled the calculation of cell activation probability. We should note that it applies limitations on what kind of network dynamics can be modeled using this approach. It would be valid when the neurons have enough time between stimuli to relax to the baseline state but would not be accurate if stimulation is applied during strong ongoing activity when the state of the neuron (including ion channel activation, etc.) changes rapidly over time.

In our analysis, we applied the same method to excitatory and inhibitory populations, which implies that all parameters of our estimation scheme (such as threshold for activating function) are the same for all cells. Since BCs have higher input resistance compared to PYs, it is possible that their experimental threshold could be lower than our estimate, which should not, however, affect our conclusions: in fact, the suppression of excitatory firing at high input current values would still hold, since it hinges on BCs spiking before PYs (which would only be enhanced by lowering the threshold).

Another potential limitation of our approach is that in our estimates we considered a homogeneous tissue. Inhomogeneity in the tissue would affect the trigger area along axons and could change activation properties in each particular cell. Since the main source of such inhomogeneity is other neurons or glia, its effect is expected to be stronger on the deep layer neurons. While the exact effects of inhomogeneity still need to be explored, we believe that the specifics of our approach can mitigate such effects. Indeed, our estimate averages across cell rotations, shifts and multiple different reconstructions to compute a probability of spiking. This implies that the effect of local changes in the tissue (driven by inhomogeneity) would likely affect the final average probability only marginally. Hence, we do not expect dramatically different estimates for cell activations to emerge from finer estimates of tissue properties.

In addition, our calculation of the electric field assumes both an infinite volume conductor and an electrical ground at infinity (see Methods). A hemi-homogenous estimate for the volume conductor (that extends infinitely away from the stimulating electrode) could technically reflect stimulation of the unirrigated exposed cortical surface more precisely, although in practice, stimulation can also be performed when the cortical surface is covered by CSF. Furthermore, the non-homogeneity and anisotropy of the conductor is ignored both in our approach and in a hemi-conductor approach. Importantly, switching our approach to hemi-homogenous would result in a uniform re-scaling of all our activation estimates [70], hence leaving unchanged our qualitative conclusion in comparing which cell types in which cortical layers are likely to activate first in response to an incoming stimulus. Moreover, although in practice an electrical ground is never actually placed at infinity, an extracranial plate on an arm or leg is a common clinical placement for a reference electrode. This results in an electrical ground at infinity for all practical purposes.

The connectivity within our network model was based on the canonical model, which captures the main picture of the information flow across cortical layers [48–51], yet overlooks finer properties like the descending projections from excitatory to inhibitory neurons [48], the activity of less frequently observed interneurons (bi-tufted, neuroglia form, etc.), the fine variability of pyramidal cells within layer II/III PYs and their projections [31, 71]. Also, the network model predictions can be extended toward multi-compartment neuronal models [72], which can take into account finer structure of inhibitory targeting (e.g. tuft vs soma-targeting interneurons) [35] and explicitly model propagation of orthodromic and antidromic spikes. The exact and detailed aspects of inter-layer connections, which update the canonical model [73] are beyond the scope of our work, which focuses on calculating the probability of direct activation of cortical cells as a result of external electrical stimulation.

Our estimates are based on activating function and do not consider the voltage dynamics within axonal trees. While it would be ideal to use biophysical neuronal models with active conductances to estimate the probability of activation, the lack of experimental data on the distribution of passive and active ion channels along the membrane surface of the different cell types considered in the study prohibits such an analysis. However, theoretical work addressing axonal dynamics through multi-compartmental modeling [28] has shown that for moderate currents the activating function correlates with voltage dynamics, and predicts the activation sites within axonal elements. For very strong currents, areas along axonal arborization could be inactivated, blocking propagation of action potential along the axon [27]. In this case, our estimate could not apply. However, the blocking phenomenon arises only for relatively strong stimulation currents and typically affects elements that are very close to the electrode [26, 27]. Hence, we expect that for small stimulation currents (considered in our study) and superficial electrode configuration, the blocking phenomenon does not qualitatively change the results of our study. Overall, the details of axonal propagation dynamics are not essential to our method, since it focuses on average estimates of response for a cell type, rather than an account of stimulation-induced dynamics in a specific cell.

While our approach can be used to provide an estimate of the probability electrical stimulation directly inducing an action potential along the axon, there are many aspects of stimulation which can indirectly affect action potential induction which we did not consider in our estimates. For instance, depolarization of axon terminals can lead to calcium release and subsequent neurotransmitter release, even in the absence of action potentials [74], and bipolar cells even require strong depolarization at the terminals to spike in response to anodic stimulation [75]. Moreover, as distance to the stimulating electrode increases past the fiber’s length constant, the optimal stimulation site shifts from the axon hillock to the end of the nerve fiber. The activating function we used partially accounted for these phenomena as it is proportional to the first derivative of the extracellular potential at nerve endings rather than the second derivative. However, as we did not model neuronal dynamics or interactions in the morphological neuron models, we could not account for all aspects of stimulation. To overcome some of the pitfalls of the electrostatic models, we chose to use an empirically determined threshold for neuronal activation.

### Generalization of the method

While we applied our analysis to the microstimulation protocol by a single superficial electrode [29], our strategy can be directly generalized to a number of more complex settings by using linear summation and adjusting the calculations for the current density: multiple electrodes (as in ENIAC [29]), in-depth stimulation (as in epilepsy [8]), non-circular electrode plates (as in DCS [9]) and bi-polar electrodes (as in DBS [5–7]) can all be accounted for. Also, we assumed a generic cortical tissue volume, but the same idea can be applied to the tissue different from a canonical cortical column (e.g., hippocampus, geniculate nuclei, brain tissue damaged after traumatic brain injury or stroke) as long as enough data on reconstructed cells are available.

Since different stimulation protocols use different current waveforms, it is important to note that this approach can be generalized to the other stimulation protocols as long as an activation threshold has been experimentally measured. The type of activity elicited by stimulation can also be expanded. Our method is presented in the context of evaluating activation mediated by axonal spikes, an effect relevant for fast and strong stimulation protocols (ENIAC, DBS), but other types of stimulation could be focused on triggering subthreshold effects, such as voltage polarization at somas compared to dendrites (tDCS) [76]. To adapt the method to account for these sub-threshold effects, one would have to embed the reconstructed cells in the electric field and consider a probability of depolarization/hyperpolarization [77], taking into account the specific orientation of each element of the reconstructed cell compared to the direction of the current field.

Finally, before the insights of this study can be applied to human studies, the method must be extended to account for the charge-balanced biphasic stimulation protocols; the typical clinical standard. If the time between pulses is greater than the relative refractory period, especially at the node of Ranvier, then application of the model to this regime is relatively straightforward. This extension is one we are currently pursuing and hope to elaborate on in the future work.

### Conclusions

We introduced a generic approach to estimate probabilities of cell activation in response to external stimulation and applied it to make testable predictions regarding effects of superficial electrical microstimulation of a canonical cortical circuit. The ongoing rapid increase in publicly-available neuron reconstructions will enable increasing the precision of our analysis and its application to other brain regions and species. Our study provides an example of the utility of basic anatomical knowledge for designing models that further our understanding of how devices can affect brain function.

## Materials and methods

### Selecting cells reconstructions within available databases

In order to obtain cell reconstructions, we use publicly available resources for neuronal morphologies [77]. S1 Table in Supporting Information summarizes the datasets used in our analysis. All cell reconstructions were corrected for tissue shrinkage and aligned when necessary. The overall dataset is not homogenous, since cells were obtained from distinct experiments, which used rats of different age (ranges from P13-P36). However, for every cell type the parameters of the probability calculations were adjusted in order to account for possible differences in thickness of the cortex and cell size. Using data provided in the references (see S1 Table), we estimated approximate boundaries for each layer, and used those values in our statistical analysis to predict activation probability. The variations introduced turned out to be small or negligible (see also comparative analysis in [33]), and would not affect our main results.

### Computing the average axonal arborization for a given cell type

Averaged axonal density (Fig 2B) represents overall morphological properties of a given type of neurons (among those in S1 Table) and gives the general intuition on how a given cell type can be affected by electrical stimulation. Since different anatomical reconstructions of the neurons of the same type have slightly different axonal arborizations, we calculated on average which locations (in 3D space) an axonal arborization of each cell type tends to occupy. The axonal density was hence computed for each cell type. This was done as following. First, for a given cell type, we fixed one point within the cortical layer where we assumed the soma of this cell type was located and we used this point as the origin (center) of a 3D volume, where axis coordinates (x, y, z) correspond to width, height and depth of a cortical slice, respectively. We binned this volume with a grid, found by uniformly spacing 100 points in the x direction, by 100 points in the y direction, by 50 points in the z direction. Each specific reconstruction of a cell was placed within this 3D grid with soma centered at the origin. If a particular 3D grid unit was “occupied” by the axonal reconstruction, we would count +1 in the density calculations and if it was not, we would add 0. This was repeated for each reconstruction of the neurons of the same type. The resulting values would be very high for locations (3D grid units) in the 3D volume where many reconstructions had axons and very low for locations where only few cells showed axons. To be able to compare the estimates across cell types, and not be biased by the exact number of reconstructions which we had in each cell type, we normalized the values we obtained and had a 3D volume axonal density. To plot the results (but not in our calculations of spiking probability), we chose to average each obtained 3D density along the z-axis (the depth of the slice). The results are all shown in Fig 2B on a logarithmic scale, where the different 2D averaged volumes for different cell types have been immersed in the biological cortical layers that each cell type would be naturally found on. This representation, while not specifically used in any calculation that follows, is useful to interpret our results on the activation probability and illustrates why different cell types are differentially affected by current stimulation.

To answer this question, we need to study how, on average, the axonal arbors of different cell types are laid out in the cortical tissue. Reconstructing with any precision a specific and complete 3D volume of cortical tissue is yet impossible and would introduce a strong limitation in our estimates by being too sensitive to the specifics of the very tissue reconstructed. Still, multiple databases containing the detailed reconstructions of different cell types from different preparations are available[77]. Thus, we propose a new method to build for each cell type an approximation of a volume distribution of its axonal arborization, by taking advantage of the large datasets available on the specific anatomy of different cortical cell types.

Fig 2A shows an example of the reconstructions for the different cell types we considered in this study: pyramidal cells (PYs), excitatory spiny stellate cells (SCs) from layer IV, basket cells (BCs), Martinotti cells (MCs) and bi-tufted interneurons. There are two types of PYs in layer V: slender-tufted neurons from layer Va [31, 32] and thick-tufted cells from layer Vb [33]. BCs include three subtypes according to the classification proposed in recent experimental works[34, 35]: large, nest and small basket cells. According to the canonical cortical microcircuit model[48–51], PYs and SCs from layers IV and Va receive input from thalamus and then innervate superficial layers, providing an incoming flow of information into cortical column [31, 32]. In turn, thick-tufted PYs (Vb) integrate the overall activity within and across columns, both neighboring and distant [32, 78], and project their output to subcortical regions [33].

Interneurons in cortex are very diverse in their morphology and functionality [35]. BCs constitute about 50% of all inhibitory cells in cortex and form the primary source of lateral inhibition within layers, targeting somas and/or proximal dendrites of PYs [34]. MCs comprise another significant fraction of interneurons, which can form cross-layer as well as cross-columnar inhibitory connections. These cells have a specific structure, with dense axonal arborization in layer I where they inhibit tuft and proximal dendrites of PYs from all layers [45]. MCs are numerous especially in infragranular layers [35], where they are also known to specifically target basal dendrites of excitatory neurons from layer IV [45]. In cortex, there are several other classes of interneurons which are found in fewer numbers: layer I interneurons, bipolar, double bouquet and bi-tufted cells [35, 79]. Our analysis includes bi-tufted and layer I interneurons, as representative examples (in the context of our study) of this dendrite-targeting class of interneurons. Our population of reconstructed layer I interneurons contains 3 distinct subclasses (classification from [79]): small, horizontal and descending interneurons.

Looking across multiple single-cell reconstructions for the same cell type, we can now design an average profile of the probability that a cell axonal arborization would occupy a given volume across layers (details on the procedure are introduced Methods). These averages are shown in Fig 2B, in logarithmic scale to emphasize the details of the differences across shapes. All types of excitatory cells, except thick-tufted PYs from layer Vb, have relatively dense axonal arborization in the top layer, which is reached by the strongest current density during surface-placed electrode stimulation (Fig 1). As for the interneurons, BCs axonal arborizations are largely contained within the layer occupied by their soma, while Martinotti and bi-tufted cells show axons with a wider vertical span and a large footprint in the top layer (Fig 2B). The axonal density of layer I cells is mainly confined in the top layer with small traces towards layer II, due to so-called descending interneurons [79].

### Estimating electric potential: A single electrode

Our problem setup is a squared electrode (side 150 μm) placed on the cortical surface (S1 Fig). The current range is 0–150 μA and duration is 200*μ*s. Overall, these parameters match common experimental settings [29]. Note that for stimulation protocols that place the current source further from cortical surface, the current would have to pass through other tissues, such as skull, dura, arachnoid or pia maters before reaching the cortical layers. In such cases, our analysis would have to take into account potential capacitive properties, which effectively act as frequency filters, and current diffusion [80]. To estimate the effect of stimulation on the tissue, we found the electrical field potential of our current source, based on the shape of the electrode and the total current injected into the tissue. Assuming that the current is uniform across the electrode surface, our source represents a homogeneous square electrode, and the resulting electric field potential can be calculated using the following expression (see S1 Text, Estimating electric potential: a single electrode)

$$\Phi (X,Y,Z)=\frac{\rho_{e}I}{4\pi A^{2}}\iint_{-A/2}^{A/2}\frac{dxdy}{\sqrt{{\left(X-x\right)}^{2}+{\left(Y-y\right)}^{2}+Z^{2}}}$$ (1)

Here *I* denotes net current, *ρ*_*e*_ is extracellular resistivity and A is the length of the square electrode edge (Fig 1A). In our analysis, we use A = 150 μm and net current I is in the range [0, 150] μA.

It should be emphasized that throughout this study we assume an infinite homogenous medium as a boundary condition when calculating the effects of electrical stimulation. Our infinite-medium approximation ignores a number of real-world components that influence the current field, such as a brain-to-air boundary condition and intermediate material between the electrode plate and the brain (dura mater, pia mater, etc.). However, we estimate all these factors to contribute to scaling the exact value of the field, without changing the overall very fast trend of current field decay with depth in cortical tissue. Since our work aims to estimate the relative probability of activation across the different cell populations and the different layers, re-scaling factors would only affect the exactness of our quantitative predictions, which might need rescaling when compared to empirical measurements, but would not affect the overall qualitative predictions that we find with our analysis. Since we are concerned with the *relative* proportion of activation among cell types which results from electrical stimulation, this simplification is justified, because considering the case of a hemi-homogenous medium, for instance, would only introduce a scaling factor of 2 [81] on the current field estimate, and would not change the overall trend (e.g. scaling with depth) of the current field in which cells are immersed.

### Estimating the activating function

According to one-dimensional cable theory the dynamics of transmembrane voltage of axonal segments can be computed as follows:

$$\frac{c_{m}dV_{i}}{dt}=\left[\frac{d}{4\rho_{i}}(\frac{V_{i-1}-2V_{i}+V_{i+1}}{\Delta x^{2}}+\frac{\Phi_{i-1}-2\Phi_{i}+\Phi_{i+1}}{\Delta x^{2}})-\sum I_{n}^{ion}\right]$$ (2)

here *V*_*i*−1_,*V*_*i*_,*V*_*i*+1_ denote transmembrane voltages of the neighboring axonal compartments (sub index denotes number of the compartment), *c*_*m*_ = 1*μF*/*cm*^2^ is a capacitance of membrane per square unit area, *d* stands for diameter of the axon (typically between 10*μm* and 1 *μm*), *ρ*_*i*_ = 300Ω∙*cm* is a resistivity of axoplasm, and Δ*x* is a discretization parameter that defines length of the compartment. The term $\sum I_{n}^{ion}$ describes the sum of intrinsic ionic currents such as fast potassium and sodium currents for spike generation, leak currents and others. As one can see, the effective transmembrane current, which arises due to extracellular electrical stimulation is described by the term

$$f=\frac{d}{4\rho_{i}}\frac{\Phi_{i-1}-2\Phi_{i}+\Phi_{i+1}}{\Delta x^{2}}$$ (3)

where Φ_*i*_,Φ_*i*−1_,Φ_*i*+1_ stand for extracellular potentials in the vicinity of axonal compartments. In fact, *f* represents activating function [26, 28] and in the limit Δ*x*→0 can be written as $f=\frac{d}{4\rho_{i}}\frac{\partial^{2}\Phi }{\partial x^{2}}$ (here *x* axis represents the direction of axonal fiber) where *d* is the diameter of the axon, and *ρ*_*i*_ is the resistivity of the axoplasm. We use the activating function (computed piecewise along reconstructed axons) to estimate the probability of axonal activation. Since we compute the activating function in each small compartment composing an entire axonal arborization, jitter in the edges of the anatomical reconstruction could introduce numeric noise in our calculation. To minimize this issue, we estimate the direction of each axonal component (the mini segment forming a compartment in the reconstruction) using the position in space of neighboring compartments up to 10 microns away. The estimated direction is then crucial to compute the activating function, which, by definition, is calculated along the axonal element direction.

As shown by Rattay [28], the activating function is a powerful tool for analyzing the effect of electrical stimulation on neuronal fibers, since it provides putative activation and suppression zones along the fibers. In case of a horizontal axon receiving anodal stimulation, the activating function along the axon shows that stimulation has a hyperpolarizing effect in the area right below the electrode (S1D Fig, blue area) and slightly depolarizing on portions of the fiber further to the sides (red areas). The distance of the axonal fiber from the electrode also influences the effect of stimulation, as shown in S1E Fig. The interplay of fiber orientation and placement in space is also shown in S1F Fig, which presents the activating function *f*(*X*,*Y*,*Z*) for vertically (f) oriented axons. In S1E and S1F Fig, the color code emphasizes that the area below the electrode has a hyperpolarizing effect on horizontal fibers but a depolarizing effect on vertical ones. The spatial/orientation selectivity of the hyperpolarization/depolarization effect is still present when considering cathodal rather than anodal stimulation, with the caveat that, since the activating function would be reversed, the areas of depolarization/hyperpolarization would switch roles compared to S1E and S1F Fig.

### Estimating the activating function threshold

To integrate the anatomical data and the estimated activating functions, we need to identify when the activating function is capable to trigger a response in the neuron. To compute such threshold, we matched the experimentally measured current-distance relationship leading to direct activation of cortical cells by the depth electrode [36, 43] (see S2 Fig). Specifically, the experimental data we aim to match define a value of the threshold injected current *I*, which one has to apply to induce a threshold effective current *f* at the initial segment (located at distance *d* from the electrode). We used a 200 *μ*s duration stimulus pulse, typical of empirical *in vivo* microstimulation experiments [36, 43]. In S2A Fig, we show a representation of the *in vivo* experiment, which we mirrored in our model. Depth electrode (modeled as a point source of current) was placed near a cell body of a reconstructed pyramidal neuron from layer II/III. Using Eqs 2 and 3, we computed the activation current *f* at the axon initial segment (since the experimental data was focused on orthodromic activation), for different values of stimulation current *I* and distances *d*.

We found that for all fixed values *f* = *Const* the resulting relation *I*(*d*) had a characteristic quadratic form, which qualitatively resembled experimental dependences of the threshold activation current on distance [36, 43]. By choosing *f* = *f*_*th*_ = 3 *pA*/*μm*^2^, we perfectly recovered the experimentally observed current-distance relation (S2B Fig, compare with Fig 8B in [36]). Consistent with experimental data [36, 37, 43], we found that when the electrode was placed at a depth close to the soma (vertical coordinate in S2A Fig) we were able to find *f*_*th*_ only for cathodal current, while anodal current could not induce any depolarizing effect on the axonal initial segment. We will use this threshold value *f*_*th*_ to define the activation probability of axonal segments.

### Computing the average probability of activation

Our computations of the averaged probability of activation are based on the calculation of the activating function and the trigger area. The trigger area comprises axonal segments with sufficiently high (above 3 *pA*/*μm*^2^) values of activating function, which can initiate axonal action potential in unmyelinated segments of axons (e.g. nodes of Ranvier). The threshold was defined based on comparison to experimentally recorded current-distance relation for pyramidal cells (see section Results for details). Note that this threshold was computed for axon initial segment, but we used the same value for nodes of Ranvier. For unmyelinated fibers we assumed 20-fold larger threshold, since their excitability is lower due smaller concentration of sodium channels. To compute the averaged probability for a certain class of cells (S1 Table) located at distance R_0_ from the electrode we applied the following steps:

- We took one of the anatomical reconstructions and placed it at a distance R_0_ from the electrode at a certain depth Z within the layer that the cell belongs to.
- We computed activating function *f* for axonal segments of the reconstructed cell. Next, the function was evaluated against the threshold (3 *pA*/*μm*^2^ for myelinated and 60 *pA*/*μm*^2^ for unmyelinated fibers). The segments, which possess large (above threshold) activating function were marked as a trigger area, whose elements may initiate axonal response.
- We then transformed length of the trigger area *L* into probability of spiking. For myelinated fibers, the trigger area should contain at least one node of Ranvier to initiate axonal response. To find an activation probability, we discretize the trigger area into small segments of length *k = 1 μm* which is a typical length of a node of Ranvier. Note that the probability that a given segment is a node of Ranvier can be approximated as a ratio *p*_*n*_ = *k*/*D* where D denotes mean internodal distance (we used 100 *μm* in all estimations). Next, the probability that axonal fiber of length L does not contain any nodes of Ranvier can be approximated as (1−*p*_*n*_)^*N*^. Here N = L /k denotes the number of segments of length k that can fit into a fiber of total length *L*. Hence, the overall probability p of response for myelinated axon can be estimated as $p=1-(1-p_{n}{)}^{N}=1-(\frac{D-k}{D}{)}^{L/k}$. For unmyelinated fibers, whose entire membrane is exposed to extracellular space, we assumed binary dependence of probability on *L*: any *L>0* (presence of trigger area) produced activation, while absence of trigger area (*L = 0*) meant no activation.
- Steps (a-c) were repeated for various locations (different Z) and for various orientations of the cell. Note that cells tend to grow towards the surface and occupy a significant area in layer I [33]. Therefore, in our calculations the depth coordinate Z varied in the range [*Z*_min_, *Z*_min_ + *cL*_*s*_], where *Z*_min_ is a minimal possible depth of the soma, *L*_*s*_ is a layer size, and *c* is a coefficient (0.1 for all cells except basket cell, for which *c = 0*.*4*). The minimal depth for a cell soma *Z*_min_ is given by the upper boundary of the cell’s layer for cell types that are compact (e.g. basket cells in infragranular layers, whose arborization does not reach the surface). For large cells, which show arborizations that can reach the cortical surface (e.g. all pyramidal cells), the *Z*_min_ value is taken as the length from the soma to the highest point in ascending arbor, whether such branch is axonal or dendritic.
- Combining the results from the above step we have an overall probability that a given cell reconstruction would be activated.
- Steps (a) to (d) were repeated for every cell reconstruction from the pool of available neurons. The probability was calculated averaging across all cells, to represent the overall likelihood of axonal activation for a given cell class at distance R_0_ from the electrode.

### Computational model of the cortical circuit: Rationale, equations and parameters

#### Rationale

The network model represents a cortical column, which contains pyramidal (PY), spiny stellate (SC), basket (BC) and Martinotti (MC) cells from layers II-V. Note that there is a large diversity of different types of interneurons in the cortex [35], but we restrict ourselves to the most common [35] and the most important types in the context of our study. BCs constitute about 50% of all interneurons in the cortex and form a major source of lateral inhibition within the layers [35]. MCs are likely to be activated in all layers due to their specific form of axonal arborization. Moreover, MCs comprise a significant part of all interneurons in infragranular layers [35, 45] where they specifically target excitatory cells in layer IV [45].

Excitatory neurons (PYs and SCs) [82] and inhibitory MCs [35] were modeled as regular spiking cells with spike rate adaptation, whereas inhibitory BCs were modeled as fast spiking cells. All interneurons had lower leak current, which resulted in a higher responsiveness in comparison to the excitatory cells [52]. Cell dynamics was governed by Hodgkin-Huxley-type kinetics, which includes fast Na^+^-K^+^ spike generating mechanism (for all types of cells), high-threshold activated Ca^2+^ current (for PY and SCs) and slow calcium-dependent potassium (AHP) current (for regular spiking cells).

#### Equations

The membrane potential is governed by the following equation [83]:

$$C_{m}\frac{dV_{i}}{dt}=I_{ion}(V_{i})+I_{i}^{syn}+I_{i}^{ext}+\eta \xi_{i};$$ (4)

The ionic currents *I*_*ion*_(*V*_*i*_), which are responsible for intrinsic cells dynamics, read:

$$I_{ion}(V_{i})=g_{Na}m_{i}^{3}h_{i}(V_{Na}-V_{i})+g_{K}n_{i}^{4}(V_{K}-V_{i})+g_{L}(V_{L}-V_{i})+g_{Ca}\frac{V_{Ca}-V_{i}}{1+exp(-(V_{i}-V_{th})/V_{shp})}+g_{ahp}\frac{c_{i}}{c_{i}+k_{d}}(V_{K}-V_{i})$$ (5)

The gating variables *m*_*i*_, *n*_*i*_, *h*_*i*_ evolve according to:

$$\frac{dx_{i}}{dt}=\alpha_{x}(V_{i})(1-x_{i})-\beta_{x}(V_{i})x_{i},$$ (6)

where *x*_*i*_ is one of gating variables. The functions *α*_*x*_(*V*) and *β*_*x*_(*V*) are:

$$\alpha_{m}(V)=\frac{0.32(54+V)}{1-exp(-0.25(V+54))},$$ (7)

$$\beta_{m}(V)=\frac{0.28(V+27)}{\exp\left(0.2(V+27)\right)-1},$$ (8)

$$\alpha_{h}=0.128\;\exp\left(-\frac{50+V}{18}\right),$$ (9)

$$\beta_{h}=\frac{4}{1+exp(-0.2(V+27))},$$ (10)

$$\alpha_{n}=\frac{0.032(V+52)}{1-exp(-0.2(V+52))},$$ (11)

$$\beta_{n}=0.5\;\exp\left(-\frac{57+V}{40}\right).$$ (12)

Calcium concentration *c*_*i*_ obeys the following equation:

$$\frac{dc_{i}}{dt}=-\alpha_{Ca}g_{Ca}\frac{V_{i}-V_{Ca}}{1+\exp\left(-\frac{V_{i}-V_{th}}{V_{shp}}\right)}-\frac{c_{i}}{\tau_{Ca}}$$ (13)

and governs calcium-dependent hyperpolarizing potassium current

$$I_{AHP}=g_{ahp}\frac{c_{i}}{c_{i}+k_{d}}(V_{K}-V_{i}),$$ (14)

which is responsible for spike-frequency adaptation. The synaptic input was modeled according to:

$$I_{i}^{syn}=\sum_{j}G_{ij}^{exc}s_{j}^{exc}(V_{syn}^{exc}-V_{i})+\sum_{j}G_{ij}^{inh}s_{j}^{inh}(V_{syn}^{inh}-V_{i})$$ (15)

where synaptic variables $s_{j}^{exc,inh}$ are governed by the following equation:

$$\frac{ds_{j}}{dt}=\alpha_{s}^{exc,inh}S(V_{j})(1-s_{j}^{exc,inh})-\beta_{s}^{exc,inh}V_{j}$$ (16)

The function *S*(*V*) reads:

$$S(V)=\frac{1}{1+exp(-100(V-20))}.$$ (17)

The term *I*_*i*_^*ext*^ is introduced in the model simulations as the means to induce spiking in our reduced neuron models in proportion to the probabilities predicted using the activating function. Thus, our network model assumes that cells are led to spike by the incoming current delivered by the electrode with probabilities found using their axonal reconstructions. However, to effectively cause that spiking in the network we needed to trigger spikes in each cell. *I*_*i*_^*ext*^ has a different value for each cell and is tuned so that members of a given cell type show a probability of spiking at the onset of the simulation as found by our estimates based on the activating function. It is important to note that *I*_*i*_^*ext*^ is not equal to the current magnitude given by the electrode but is instead a parameter tuned so that the population of cells is activated in proportion to our estimated probabilities of spiking. The term *ηξ*_*i*_(*t*) corresponds to the fluctuations in the afferent input (representing spontaneous background activity) which are given by a white noise process (*ξ*) with standard deviation *η*. All the model parameters are listed in S2 Table (unless specified in the description of simulations), and the network structure and connectivity are described in S3 Table and S4 Table respectively.

Cells were synaptically coupled by excitatory (AMPA) and inhibitory (GABA_A_) connections. The structure of synaptic connections represented a random graph. The strength and probability of connections depended on the layer and the cell type, resembling structure of a canonical cortical circuit [48]. Each layer contained relatively strong recurrent excitatory (PY-to-PY and PY-to-BC) and lateral inhibitory (BC-to-PY) connections. According to canonical architecture, PYs within layers II/III were driven by strong connections from layers IV and Va [31, 48, 71]. Excitatory cells from layers IV and Va (slender-tufted PY) were strongly coupled through recurrent excitatory connections within the layers [32, 82, 84]. In addition, PYs within layer Va received moderate excitatory input from layer IV [32]. Inhibitory lateral connections from interneurons to PY were more intense and strong than excitatory ones[85]. BCs formed local connections that project to excitatory cells within their own layers [35]. In accordance with experimental studies [45], MCs from layer IV projected to excitatory cells from layer IV and II/III, whereas layer V MCs contacted excitatory cells from layer IV.

## Supporting information

S1 Text Description of the method used to estimate electric potential for a single electrode. This text contains a more, detailed and technical description of the computational methods used to estimate the electric potential for a single electrode and describes how the data presented in S1 Fig were generated.
(PDF)

S1 Fig Electric potential under electrode plate and its effect on axonal fibers (the case of anodal stimulation is shown). **(a)** Schematic representation of the electrode in the coordinate system (X,Y,Z). Electrode is located on the surface (gray), center of the coordinate system corresponds to the center of the electrode. **(b)** Electric potential Φ(*X*,*Y*,*Z*) on the plane Y = 0 (marked by red in panel (a)). **(c)** Comparison of the electric potential induced by point source (Eq (4), dashed curve) and finite-size square plate (Eq (3), solid curve) at varying depth Z and fixed X = Y = 0. **(d)** Top panel shows schematic representation of the electrode (red) and horizontally oriented axon (green) on (X,Z) plane (Y = 0). Bottom panels show potential Φ(x) and activating function ∂^2^Φ(x)/∂*x*^2^ along axonal fiber (anodal stimulation). **(e,f)** Activating function for horizontally **(e)** and vertically **(f)** oriented fibers as a function of coordinates X,Z on the plane Y = 0. Black solid curves separate areas of depolarization (red) and hyperpolarization (blue). Note that for cathodal stimulation the activating function is exactly opposite (area of depolarization and hyperpolarization are interchanged).
(TIFF)

S2 Fig Theoretical method faithfully reproduces current-distance relation observed in experiments [36] (direct activation of cell by depth electrode). **(a)** Schematic representation of depth electrode (point source of current) and pyramidal cell. Green color denotes axons, purple and blue colors show apical and basal dendrites correspondingly. **(b)** Current-distance relation for direct activation of pyramidal cells (initial segment) by depth electrode. Blue curve represents average dependence across array of different reconstructions and rotations of pyramidal cells, gray area denotes mean plus/minus standard deviation. An ensemble of 15 cells was used.
(TIFF)

S1 Table Summary of datasets with reconstructed cells. (PDF)

S2 Table Model parameters for network simulations. PY–pyramidal cell, SC–spiny stellate cell, MC–Martinotti cell, BC–basket cell.
(PDF)

S3 Table Structure of the network. PY–pyramidal neurons, BC–basket cells, SC–excitatory spiny stellate cells, MC–Martinotti cells.
(PDF)

S4 Table Connectivity within the network. PY–pyramidal neurons, BC–basket cells, SC–excitatory spiny stellate cells, MC–Martinotti cells.
(PDF)

## References

[1] Tehovnik EJ, Lee K. The dorsomedial frontal cortex of the rhesus monkey: topographic representation of saccades evoked by electrical stimulation. Exp Brain Res. 1993;96(3):430–42. 10.1007/bf00234111 . 8299745

[2] Graziano MS, Taylor CS, Moore T. Complex movements evoked by microstimulation of precentral cortex. Neuron. 2002;34(5):841–51. . 12062029 10.1016/s0896-6273(02)00698-0

[3] Britten KH, van Wezel RJ. Electrical microstimulation of cortical area MST biases heading perception in monkeys. Nat Neurosci. 1998;1(1):59–63. 10.1038/259 . 10195110

[4] Halgren E, Walter RD, Cherlow DG, Crandall PH. Mental phenomena evoked by electrical stimulation of the human hippocampal formation and amygdala. Brain. 1978;101(1):83–117. 10.1093/brain/101.1.83 . 638728

[5] Blumenfeld Z, Brontë-Stewart H. High Frequency Deep Brain Stimulation and Neural Rhythms in Parkinson's Disease. Neuropsychol Rev. 2015;25(4):384–97. 10.1007/s11065-015-9308-7 . 26608605

[6] Papageorgiou PN, Deschner J, Papageorgiou SN. Effectiveness and Adverse Effects of Deep Brain Stimulation: Umbrella Review of Meta-Analyses. J Neurol Surg A Cent Eur Neurosurg. 2016 10.1055/s-0036-1592158 . 27642815

[7] Baizabal-Carvallo JF, Alonso-Juarez M. Low-frequency deep brain stimulation for movement disorders. Parkinsonism Relat Disord. 2016 10.1016/j.parkreldis.2016.07.018 . 27497841

[8] Nagaraj V, Lee ST, Krook-Magnuson E, Soltesz I, Benquet P, Irazoqui PP, et al Future of seizure prediction and intervention: closing the loop. J Clin Neurophysiol. 2015;32(3):194–206. 10.1097/WNP.0000000000000139 26035672 PMC4455045

[9] Hummel FC, Cohen LG. Non-invasive brain stimulation: a new strategy to improve neurorehabilitation after stroke? Lancet Neurol. 2006;5(8):708–12. 10.1016/S1474-4422(06)70525-7 . 16857577

[10] Fröhlich F, Sellers KK, Cordle AL. Targeting the neurophysiology of cognitive systems with transcranial alternating current stimulation. Expert Rev Neurother. 2015;15(2):145–67. 10.1586/14737175.2015.992782 25547149 PMC4634940

[11] Summers JJ, Kang N, Cauraugh JH. Does transcranial direct current stimulation enhance cognitive and motor functions in the ageing brain? A systematic review and meta- analysis. Ageing Res Rev. 2016;25:42–54. 10.1016/j.arr.2015.11.004 . 26607412

[12] Prehn K, Flöel A. Potentials and limits to enhance cognitive functions in healthy and pathological aging by tDCS. Front Cell Neurosci. 2015;9:355 10.3389/fncel.2015.00355 26441526 PMC4568338

[13] Savic B, Meier B. How Transcranial Direct Current Stimulation Can Modulate Implicit Motor Sequence Learning and Consolidation: A Brief Review. Front Hum Neurosci. 2016;10:26 10.3389/fnhum.2016.00026 26903837 PMC4748051

[14] Draganski B, Kherif F, Lutti A. Computational anatomy for studying use-dependant brain plasticity. Front Hum Neurosci. 2014;8:380 10.3389/fnhum.2014.00380 25018716 PMC4072968

[15] Tolias AS, Sultan F, Augath M, Oeltermann A, Tehovnik EJ, Schiller PH, et al Mapping cortical activity elicited with electrical microstimulation using FMRI in the macaque. Neuron. 2005;48(6):901–11. Epub 2005/12/21. 10.1016/j.neuron.2005.11.034 . 16364895

[16] Polanía R, Paulus W, Antal A, Nitsche MA. Introducing graph theory to track for neuroplastic alterations in the resting human brain: a transcranial direct current stimulation study. Neuroimage. 2011;54(3):2287–96. 10.1016/j.neuroimage.2010.09.085 . 20932916

[17] Popovych OV, Tass PA. Desynchronizing electrical and sensory coordinated reset neuromodulation. Front Hum Neurosci. 2012;6:58 10.3389/fnhum.2012.00058 22454622 PMC3308339

[18] Rahman A, Reato D, Arlotti M, Gasca F, Datta A, Parra LC, et al Cellular effects of acute direct current stimulation: somatic and synaptic terminal effects. J Physiol. 2013;591(10):2563–78. 10.1113/jphysiol.2012.247171 23478132 PMC3678043

[19] McIntyre CC, Savasta M, Kerkerian-Le Goff L, Vitek JL. Uncovering the mechanism(s) of action of deep brain stimulation: activation, inhibition, or both. Clin Neurophysiol. 2004;115(6):1239–48. 10.1016/j.clinph.2003.12.024 . 15134690

[20] Seidemann E, Arieli A, Grinvald A, Slovin H. Dynamics of depolarization and hyperpolarization in the frontal cortex and saccade goal. Science. 2002;295(5556):862–5. Epub 2002/02/02. 10.1126/science.1066641 . 11823644

[21] Meffin H, Tahayori B, Grayden DB, Burkitt AN. Modeling extracellular electrical stimulation: I. Derivation and interpretation of neurite equations. J Neural Eng. 2012;9(6):065005 10.1088/1741-2560/9/6/065005 . 23187045

[22] Datta A, Bansal V, Diaz J, Patel J, Reato D, Bikson M. Gyri-precise head model of transcranial direct current stimulation: improved spatial focality using a ring electrode versus conventional rectangular pad. Brain Stimul. 2009;2(4):201–7, 7.e1. 10.1016/j.brs.2009.03.005 20648973 PMC2790295

[23] Datta A, Baker JM, Bikson M, Fridriksson J. Individualized model predicts brain current flow during transcranial direct-current stimulation treatment in responsive stroke patient. Brain Stimul. 2011;4(3):169–74. 10.1016/j.brs.2010.11.001 21777878 PMC3142347

[24] Histed MH, Bonin V, Reid RC. Direct activation of sparse, distributed populations of cortical neurons by electrical microstimulation. Neuron. 2009;63(4):508–22. 10.1016/j.neuron.2009.07.016 19709632 PMC2874753

[25] Tehovnik EJ, Slocum WM. Two-photon imaging and the activation of cortical neurons. Neuroscience. 2013;245:12–25. 10.1016/j.neuroscience.2013.04.022 . 23603308

[26] Rattay F. Ways to approximate current-distance relations for electrically stimulated fibers. J Theor Biol. 1987;125(3):339–49. 10.1016/s0022-5193(87)80066-8 . 3657215

[27] Rattay F, Aberham M. Modeling axon membranes for functional electrical stimulation. IEEE Trans Biomed Eng. 1993;40(12):1201–9. 10.1109/10.250575 . 8125496

[28] Rattay F. The basic mechanism for the electrical stimulation of the nervous system. Neuroscience. 1999;89(2):335–46. 10.1016/s0306-4522(98)00330-3 . 10077317

[29] Ha BS, Akinin A, Park J, Kim C, Wang H, Maier C, et al Silicon-Integrated High-Density Electrocortical Interfaces. Proceedings of the IEEE 2016.

[30] DeFelipe J. The evolution of the brain, the human nature of cortical circuits, and intellectual creativity. Frontiers in Neuroanatomy 2011 p. 29:1–17. 21647212 10.3389/fnana.2011.00029 PMC3098448

[31] Shepherd GM, Svoboda K. Laminar and columnar organization of ascending excitatory projections to layer 2/3 pyramidal neurons in rat barrel cortex. J Neurosci. 2005;25(24):5670–9. 10.1523/JNEUROSCI.1173-05.2005 . 15958733 PMC6724876

[32] Schubert D, Kötter R, Luhmann HJ, Staiger JF. Morphology, electrophysiology and functional input connectivity of pyramidal neurons characterizes a genuine layer va in the primary somatosensory cortex. Cereb Cortex. 2006;16(2):223–36. 10.1093/cercor/bhi100 . 15872153

[33] Ramaswamy S, Markram H. Anatomy and physiology of the thick-tufted layer 5 pyramidal neuron. Front Cell Neurosci. 2015;9:233 10.3389/fncel.2015.00233 26167146 PMC4481152

[34] Wang Y, Gupta A, Toledo-Rodriguez M, Wu CZ, Markram H. Anatomical, physiological, molecular and circuit properties of nest basket cells in the developing somatosensory cortex. Cereb Cortex. 2002;12(4):395–410. 10.1093/cercor/12.4.395 . 11884355

[35] Markram H, Toledo-Rodriguez M, Wang Y, Gupta A, Silberberg G, Wu C. Interneurons of the neocortical inhibitory system. Nature reviews Neuroscience. 2004;5(10):793–807. Epub 2004/09/21. 10.1038/nrn1519 . 15378039

[36] Stoney SJ, Thompson W, Asanuma H. Excitation of pyramidal tract cells by intracortical microstimulation: effective extent of stimulating current. J Neurophysiol.1968 p. 659–69. 10.1152/jn.1968.31.5.659 5711137

[37] Ranck JB. Which elements are excited in electrical stimulation of mammalian central nervous system: a review. Brain Res. 1975;98(3):417–40. 10.1016/0006-8993(75)90364-9 . 1102064

[38] Gustafsson B, Jankowska E. Direct and indirect cells activation of nerve cells by electrical pulses applied extracellularly. J. Physiol.1976 p. 33–61. 10.1113/jphysiol.1976.sp011405 PMC1308958 940071

[39] Nowak LG, Bullier J. Axons, but not cell bodies, are activated by electrical stimulation in cortical gray matter. I. Evidence from chronaxie measurements. Exp Brain Res. 1998;118(4):477–88. 10.1007/s002210050304 . 9504843

[40] Nowak LG, Bullier J. Axons, but not cell bodies, are activated by electrical stimulation in cortical gray matter. II. Evidence from selective inactivation of cell bodies and axon initial segments. Exp Brain Res. 1998;118(4):489–500. 10.1007/s002210050305 . 9504844

[41] Porter R. FOCAL STIMULATION OF HYPOGLOSSAL NEURONES IN THE CAT. J Physiol. 1963;169:630–40. 10.1113/jphysiol.1963.sp007285 14082123 PMC1368726

[42] Swadlow HA. Monitoring the excitability of neocortical efferent neurons to direct activation by extracellular current pulses. J Neurophysiol. 1992;68(2):605–19. 10.1152/jn.1992.68.2.605 . 1527578

[43] Tehovnik EJ, Tolias AS, Sultan F, Slocum WM, Logothetis NK. Direct and Indirect Activation of Cortical Neurons by Electrical Microstimulation. J. Neurophysiol.2006 p. 512–21. 10.1152/jn.00126.2006 16835359

[44] Ford MC, Alexandrova O, Cossell L, Stange-Marten A, Sinclair J, Kopp-Scheinpflug C, et al Tuning of Ranvier node and internode properties in myelinated axons to adjust action potential timing. Nat Commun. 2015;6:8073 Epub 2015/08/25. 10.1038/ncomms9073 26305015 PMC4560803

[45] Wang Y, Toledo-Rodriguez M, Gupta A, Wu C, Silberberg G, Luo J, et al Anatomical, physiological and molecular properties of Martinotti cells in the somatosensory cortex of the juvenile rat. J Physiol. 2004;561(Pt 1):65–90. 10.1113/jphysiol.2004.073353 15331670 PMC1665344

[46] Freeman SA, Desmazières A, Fricker D, Lubetzki C, Sol-Foulon N. Mechanisms of sodium channel clustering and its influence on axonal impulse conduction. Cell Mol Life Sci. 2016;73(4):723–35. Epub 2015/10/29. 10.1007/s00018-015-2081-1 26514731 PMC4735253

[47] Tomassy GS, Berger DR, Chen HH, Kasthuri N, Hayworth KJ, Vercelli A, et al Distinct profiles of myelin distribution along single axons of pyramidal neurons in the neocortex. Science. 2014;344(6181):319–24. 10.1126/science.1249766 24744380 PMC4122120

[48] Thomson AM, West DC, Wang Y, Bannister AP. Synaptic connections and small circuits involving excitatory and inhibitory neurons in layers 2–5 of adult rat and cat neocortex: triple intracellular recordings and biocytin labelling in vitro. Cereb Cortex. 2002;12(9):936–53. 10.1093/cercor/12.9.936 . 12183393

[49] Douglas RJ, Martin KAC, Whitteridge D. A Canonical Microcircuit for Neocortex Neural Computation 1989 p. 480–8.

[50] Defelipe J, Markram H, Rockland KS. The neocortical column. Front Neuroanat. 2012;6:22 10.3389/fnana.2012.00005 ; PubMed Central PMCID: PMCPMC3383203. 22745629 PMC3383203

[51] da Costa NM, Martin KA. Whose Cortical Column Would that Be? Front Neuroanat. 2010;4:16 10.3389/fnana.2010.00016 20640245 PMC2904586

[52] Povysheva NV, Gonzalez-Burgos G, Zaitsev AV, Kröner S, Barrionuevo G, Lewis DA, et al Properties of excitatory synaptic responses in fast-spiking interneurons and pyramidal cells from monkey and rat prefrontal cortex. Cereb Cortex. 2006;16(4):541–52. 10.1093/cercor/bhj002 . 16033926

[53] Lewis PM, Ackland HM, Lowery AJ, Rosenfeld JV. Restoration of vision in blind individuals using bionic devices: a review with a focus on cortical visual prostheses. Brain Res. 2015;1595:51–73. 10.1016/j.brainres.2014.11.020 . 25446438

[54] Kuo MF, Nitsche MA. Effects of transcranial electrical stimulation on cognition. Clin EEG Neurosci. 2012;43(3):192–9. 10.1177/1550059412444975 . 22956647

[55] Stagg CJ, Nitsche MA. Physiological basis of transcranial direct current stimulation. Neuroscientist. 2011;17(1):37–53. 10.1177/1073858410386614 . 21343407

[56] Jackson MP, Rahman A, Lafon B, Kronberg G, Ling D, Parra LC, et al Animal models of transcranial direct current stimulation: Methods and mechanisms. Clin Neurophysiol. 2016;127(11):3425–54. Epub 2016/09/10. 10.1016/j.clinph.2016.08.016 ; PubMed Central PMCID: PMCPMC5083183. 27693941 PMC5083183

[57] Malerba P, Straudi S, Fregni F, Bazhenov M, Basaglia N. Using Biophysical Models to Understand the Effect of tDCS on Neurorehabilitation: Searching for Optimal Covariates to Enhance Poststroke Recovery. Frontiers in Neurology. 2017;8(58). 10.3389/fneur.2017.00058 28280482 PMC5322214

[58] Liebetanz D, Klinker F, Hering D, Koch R, Nitsche MA, Potschka H, et al Anticonvulsant effects of transcranial direct-current stimulation (tDCS) in the rat cortical ramp model of focal epilepsy. Epilepsia. 2006;47(7):1216–24. 10.1111/j.1528-1167.2006.00539.x . 16886986

[59] Merrill DR, Bikson M, Jefferys JG. Electrical stimulation of excitable tissue: design of efficacious and safe protocols. J Neurosci Methods. 2005;141(2):171–98. 10.1016/j.jneumeth.2004.10.020 . 15661300

[60] Cogan SF, Ludwig KA, Welle CG, Takmakov P. Tissue damage thresholds during therapeutic electrical stimulation. J Neural Eng. 2016;13(2):021001 Epub 2016/01/20. 10.1088/1741-2560/13/2/021001 26792176 PMC5386002

[61] RALL W. Theory of physiological properties of dendrites. Ann N Y Acad Sci. 1962;96:1071–92. 10.1111/j.1749-6632.1962.tb54120.x . 14490041

[62] Tranchina D, Nicholson C. A model for the polarization of neurons by extrinsically applied electric fields. Biophys J. 1986;50(6):1139–56. 10.1016/S0006-3495(86)83558-5 3801574 PMC1329788

[63] Berzhanskaya J, Chernyy N, Gluckman BJ, Schiff SJ, Ascoli GA. Modulation of hippocampal rhythms by subthreshold electric fields and network topology. Journal of computational neuroscience. 2013;34(3):369–89. Epub 2012/10/12. 10.1007/s10827-012-0426-4 23053863 PMC3549326

[64] HERN JE, LANDGREN S, PHILLIPS CG, PORTER R. Selective excitation of corticofugal neurones by surface-anodal stimulation of the baboon's motor cortex. J Physiol. 1962;161:73–90. 10.1113/jphysiol.1962.sp006874 13906736 PMC1359595

[65] Pollen DA. Responses of single neurons to electrical stimulation of the surface of the visual cortex. Brain Behav Evol. 1977;14(1–2):67–86. 10.1159/000125576 . 837212

[66] Manola L, Holsheimer J, Veltink P, Buitenweg JR. Anodal vs cathodal stimulation of motor cortex: a modeling study. Clin Neurophysiol. 2007;118(2):464–74. Epub 2006/12/05. 10.1016/j.clinph.2006.09.012 . 17150409

[67] Catterall WA. Localization of sodium channels in cultured neural cells. J Neurosci. 1981;1(7):777–83. . 6286901 10.1523/JNEUROSCI.01-07-00777.1981 PMC6564194

[68] Joucla S, Branchereau P, Cattaert D, Yvert B. Extracellular neural microstimulation may activate much larger regions than expected by simulations: a combined experimental and modeling study. PLoS One. 2012;7(8):e41324 Epub 2012/08/11. 10.1371/journal.pone.0041324 22879886 PMC3413686

[69] Joucla S, Yvert B. The "mirror" estimate: an intuitive predictor of membrane polarization during extracellular stimulation. Biophys J. 2009;96(9):3495–508. 10.1016/j.bpj.2008.12.3961 19413956 PMC2711410

[70] Buccino A, Kuchta M, Jaeger K, Ness T, Berthet P, Mardal K-A, et al How does the presence of neural probes affect extracellular potentials? bioRxiv. 2019:doi: 10.1101/318741. 10.1101/318741. 30703758

[71] Staiger JF, Bojak I, Miceli S, Schubert D. A gradual depth-dependent change in connectivity features of supragranular pyramidal cells in rat barrel cortex. Brain Struct Funct. 2015;220(3):1317–37. 10.1007/s00429-014-0726-8 24569853 PMC4409644

[72] Bonjean M, Baker T, Bazhenov M, Cash S, Halgren E, Sejnowski T. Interactions between core and matrix thalamocortical projections in human sleep spindle synchronization. J Neurosci. 2012;32(15):5250–63. 10.1523/JNEUROSCI.6141-11.2012 22496571 PMC3342310

[73] Dhruv NT. Rethinking canonical cortical circuits. Nat Neurosci. 2015;18(11):1538 10.1038/nn1115-1538 . 26505564

[74] Katz B, Miledi R. A study of synaptic transmission in the absence of nerve impulses. J Physiol. 1967;192(2):407–36. Epub 1967/09/01. 10.1113/jphysiol.1967.sp008307 4383089 PMC1365564

[75] Rattay F, Bassereh H, Fellner A. Impact of Electrode Position on the Elicitation of Sodium Spikes in Retinal Bipolar Cells. Sci Rep. 2017;7(1):17590 Epub 2017/12/16. 10.1038/s41598-017-17603-8 29242502 PMC5730545

[76] Bikson M, Rahman A, Datta A. Computational models of transcranial direct current stimulation. Clin EEG Neurosci. 2012;43(3):176–83. Epub 2012/09/08. 10.1177/1550059412445138 . 22956646

[77] Ascoli GA, Donohue DE, Halavi M. NeuroMorpho.Org: a central resource for neuronal morphologies. J Neurosci. 2007;27(35):9247–51. 10.1523/JNEUROSCI.2055-07.2007 . 17728438 PMC6673130

[78] Schubert D, Staiger JF, Cho N, Kötter R, Zilles K, Luhmann HJ. Layer-specific intracolumnar and transcolumnar functional connectivity of layer V pyramidal cells in rat barrel cortex. J Neurosci. 2001;21(10):3580–92. . 11331387 10.1523/JNEUROSCI.21-10-03580.2001 PMC6762473

[79] Muralidhar S, Wang Y, Markram H. Synaptic and cellular organization of layer 1 of the developing rat somatosensory cortex. Front Neuroanat. 2013;7:52 10.3389/fnana.2013.00052 24474905 PMC3893566

[80] Nunez PL, Srinivasan R. Electric Fields of the Brain: The Neurophysics of EEG. 2 ed: Oxford University Press; 2005.

[81] Buccino AK, Miroslav; Jaeger Karoline; Ness Torbjorn; Mardal Kent-Andre; Cauwenberghs Gert; Tveito Aslak. Can the presence of neural probes be neglected in computational modeling of extracellular potentials? bioRxiv. 2018 10.1101/318741.

[82] Schubert D, Kötter R, Zilles K, Luhmann HJ, Staiger JF. Cell type-specific circuits of cortical layer IV spiny neurons. J Neurosci. 2003;23(7):2961–70. . 12684483 10.1523/JNEUROSCI.23-07-02961.2003 PMC6742105

[83] Traub RD, Wong RK, Miles R, Michelson H. A model of a CA3 hippocampal pyramidal neuron incorporating voltage-clamp data on intrinsic conductances. J Neurophysiol. 1991;66(2):635–50. 10.1152/jn.1991.66.2.635 . 1663538

[84] Staiger JF, Flagmeyer I, Schubert D, Zilles K, Kötter R, Luhmann HJ. Functional diversity of layer IV spiny neurons in rat somatosensory cortex: quantitative morphology of electrophysiologically characterized and biocytin labeled cells. Cereb Cortex. 2004;14(6):690–701. 10.1093/cercor/bhh029 . 15054049

[85] Holmgren C, Harkany T, Svennenfors B, Zilberter Y. Pyramidal cell communication within local networks in layer 2/3 of rat neocortex. J Physiol. 2003;551(Pt 1):139–53. 10.1113/jphysiol.2003.044784 12813147 PMC2343144

