# Supplementary material for: Selective recruitment of cortical neurons by electrical stimulation
Source: PLoS Comput Biol. 2019 Aug 26;15(8):e1007277. doi: 10.1371/journal.pcbi.1007277 (PMC6742409; doi:10.1371/journal.pcbi.1007277)
Supplement: S1 Text — This text contains a more, detailed and technical description of the computational methods used to estimate the electric potential for a single electrode and describes how the data presented in S1 Fig were generated. (PDF) [file pcbi.1007277.s001.pdf]

Supporting Information:

Selective Recruitment of Cortical Neurons by Electrical Stimulation

Maxim Komarov[1], Paola Malerba[1], Ryan Golden[1], Paul Nunez[2], Eric Halgren[3], Maxim Bazhenov[1]

Estimating electric potential: a single electrode.

Below we assume the current delivered by the stimulation electrode (*I*) a fixed amount. We consider a Cartesian coordinate system (*X,Y,Z*) centered at the electrode, where the *Z* axis is oriented perpendicular to the electrode surface and goes in depth across cortical layers; the axes X and Y are oriented parallel to the surface and electrode plate (see S1a Fig.). To a first approximation, we assume that the current *I* across the insulating layer of the electrode is uniform over the electrode surface. Each infinitesimally small element of the electrode *dS* then produces a current source

$$dI = \frac{IdS}{S} = \frac{I}{S}dxdy \quad (1)$$

Where *S* is the electrode surface and *dS = dxdy* is the infinitesimal electrode element. Here, the pair *(x, y)* locates the current source on the surface of the squared electrode (where *Z=0*). The elemental current source produces a tissue potential at distance *L* from the source given by

$$d\Phi = \frac{\rho_e I}{4\pi S}\frac{dxdy}{L} \quad (2)$$

where ρ_e is the resistivity of the tissue, $S = A^2$ is the surface area of the electrode, *A* is the size of the electrode and *I* is the total current generated by the electrode. Note that the distance can be expressed as $L = \sqrt{(X-x)^2 + (Y-y)^2 + Z^2}$, where the capitalized letters identify coordinates in the cortical volume, and the lowercase letters mark coordinates within the electrode surface. Hence, the potential in the Cartesian coordinate system (*X,Y,Z*) is given by

$$\Phi(X,Y,Z) = \frac{\rho_e I}{4\pi A^2}\iint_{-A/2}^{A/2}\frac{dxdy}{\sqrt{(X-x)^2+(Y-y)^2+Z^2}} \quad (3)$$

The integral form of equation (3) is due to the need to compute the contribution of the entire electrode surface area *A* as a source of electrical current. However, at large distances the finite size of the electrode has a nominal effect on the electrical field. Indeed, the rapid decay of potential with respect to distance from the source is evident (S1b Fig). Note that for tissue locations further away from the electrode plate, the point-source approximation provides the following simplified expression to compute the electrical potential field,

$$\Phi(X,Y,Z) = \frac{\rho_e I}{4\pi\sqrt{X^2+Y^2+Z^2}}. \quad (4)$$

This estimate well approximates our exact solution (in equation (1)) at a distance from the electrode, however it fails for locations right below the electrode plate, where the finite size of the electrode should be taken into account (S1c Fig.). This distinction is particularly relevant when considering the potential field in cortical layers I and II, immediately below the surface, which are also the most affected by stimulation due to the decay of Φ with depth. Hence, we emphasize that close to the electrode, the point-source approximation does not yield an estimate that can be used to predict precisely the effect of cortical stimulation.

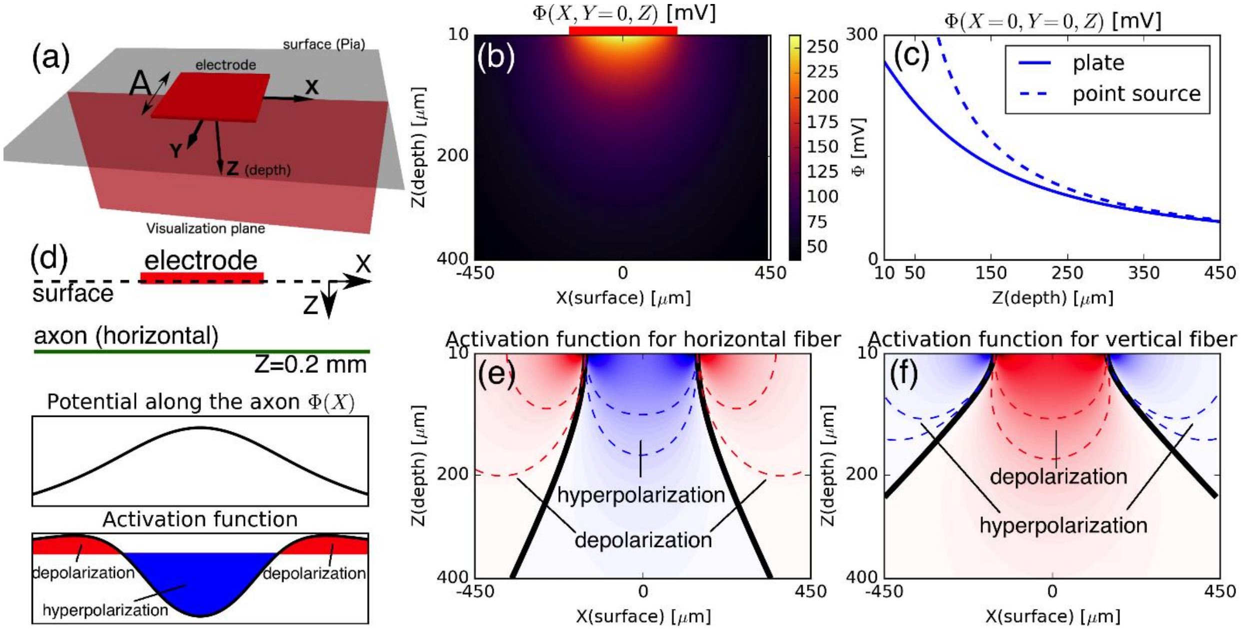

S1 Fig. Electric potential under electrode plate and its effect on axonal fibers (the case of anodal stimulation is shown). (a) Schematic representation of the electrode in the coordinate system (X,Y,Z). Electrode is located on the surface (gray), center of the coordinate system corresponds to the center of the electrode. **(b)** Electric potential $\Phi(X, Y, Z)$ on the plane Y=0 (marked by red in panel (a)). **(c)** Comparison of the electric potential induced by point source (eq. (4), dashed curve) and finite-size square plate (eq. (3), solid curve) at varying depth Z and fixed X=Y=0. **(d)** Top panel shows schematic representation of the electrode (red) and horizontally oriented axon (green) on (X,Z) plane (Y=0). Bottom panels show potential Φ(x) and activating function $\partial^2\Phi(\mathrm{x})/\partial x^2$ along axonal fiber (anodal stimulation). **(e,f)** Activating function for horizontally **(e)** and vertically **(f)** oriented fibers as a function of coordinates X,Z on the plane Y=0. Black solid curves separate areas of depolarization (red) and hyperpolarization (blue). Note that for cathodal stimulation the activating function is exactly opposite (area of depolarization and hyperpolarization are interchanged).

References

1. Ha BS, Akinin A, Park J, Kim C, Wang H, Maier C, et al. Silicon-Integrated High-Density Electrocortical Interfaces. Proceedings of the IEEE2016.
2. Nunez PL, Srinivasan R. Electric Fields of the Brain: The Neurophysics of EEG. 2 ed: Oxford University Press; 2005.
3. Buccino AK, Miroslav; Jaeger, Karoline; Ness, Torbjorn; Mardal, Kent-Andre; Cauwenberghs, Gert; Tveito, Aslak. Can the presence of neural probes be neglected in computational modeling of extracellular potentials? bioRxiv. 2018. doi: https://doi.org/10.1101/318741.
4. Ascoli GA, Donohue DE, Halavi M. NeuroMorpho.Org: a central resource for neuronal morphologies. J Neurosci. 2007;27(35):9247-51. doi: 10.1523/JNEUROSCI.2055-07.2007. PubMed PMID: 17728438.
5. Shepherd GM, Svoboda K. Laminar and columnar organization of ascending excitatory projections to layer 2/3 pyramidal neurons in rat barrel cortex. J Neurosci. 2005;25(24):5670-9. doi: 10.1523/JNEUROSCI.1173-05.2005. PubMed PMID: 15958733.
6. Schubert D, Kötter R, Luhmann HJ, Staiger JF. Morphology, electrophysiology and functional input connectivity of pyramidal neurons characterizes a genuine layer va in the primary somatosensory cortex. Cereb Cortex. 2006;16(2):223-36. doi: 10.1093/cercor/bhi100. PubMed PMID: 15872153.
7. Ramaswamy S, Markram H. Anatomy and physiology of the thick-tufted layer 5 pyramidal neuron. Front Cell Neurosci. 2015;9:233. doi: 10.3389/fncel.2015.00233. PubMed PMID: 26167146; PubMed Central PMCID: PMCPMC4481152.
8. Wang Y, Gupta A, Toledo-Rodriguez M, Wu CZ, Markram H. Anatomical, physiological, molecular and circuit properties of nest basket cells in the developing somatosensory cortex. Cereb Cortex. 2002;12(4):395-410. PubMed PMID: 11884355.
9. Markram H, Toledo-Rodriguez M, Wang Y, Gupta A, Silberberg G, Wu C. Interneurons of the neocortical inhibitory system. Nature reviews Neuroscience. 2004;5(10):793-807. Epub 2004/09/21. doi: 10.1038/nrn1519. PubMed PMID: 15378039.
10. Thomson AM, West DC, Wang Y, Bannister AP. Synaptic connections and small circuits involving excitatory and inhibitory neurons in layers 2-5 of adult rat and cat neocortex: triple intracellular recordings and biocytin labelling in vitro. Cereb Cortex. 2002;12(9):936-53. PubMed PMID: 12183393.
11. Douglas RJ, Martin KAC, Whitteridge D. A Canonical Microcircuit for Neocortex Neural Computation1989. p. 480-8.
12. Defelipe J, Markram H, Rockland KS. The neocortical column. Front Neuroanat. 2012;6:22. doi: 10.3389/fnana.2012.00005. PubMed PMID: 22745629; PubMed Central PMCID: PMCPMC3383203.
13. da Costa NM, Martin KA. Whose Cortical Column Would that Be? Front Neuroanat. 2010;4:16. doi: 10.3389/fnana.2010.00016. PubMed PMID: 20640245; PubMed Central PMCID: PMCPMC2904586.

14. Schubert D, Staiger JF, Cho N, Kötter R, Zilles K, Luhmann HJ. Layer-specific intracolumnar and transcolumnar functional connectivity of layer V pyramidal cells in rat barrel cortex. J Neurosci. 2001;21(10):3580-92. PubMed PMID: 11331387.
15. Wang Y, Toledo-Rodriguez M, Gupta A, Wu C, Silberberg G, Luo J, et al. Anatomical, physiological and molecular properties of Martinotti cells in the somatosensory cortex of the juvenile rat. J Physiol. 2004;561(Pt 1):65-90. doi: 10.1113/jphysiol.2004.073353. PubMed PMID: 15331670; PubMed Central PMCID: PMCPMC1665344.
16. Muralidhar S, Wang Y, Markram H. Synaptic and cellular organization of layer 1 of the developing rat somatosensory cortex. Front Neuroanat. 2013;7:52. doi: 10.3389/fnana.2013.00052. PubMed PMID: 24474905; PubMed Central PMCID: PMCPMC3893566.
17. Stoney SJ, Thompson W, Asanuma H. Excitation of pyramidal tract cells by intracortical microstimulation: effective extent of stimulating current. J Neurophysiol.1968. p. 659-69.
18. Staiger JF, Bojak I, Miceli S, Schubert D. A gradual depth-dependent change in connectivity features of supragranular pyramidal cells in rat barrel cortex. Brain Struct Funct. 2015;220(3):1317-37. doi: 10.1007/s00429-014-0726-8. PubMed PMID: 24569853; PubMed Central PMCID: PMCPMC4409644.
19. Staiger JF, Flagmeyer I, Schubert D, Zilles K, Kötter R, Luhmann HJ. Functional diversity of layer IV spiny neurons in rat somatosensory cortex: quantitative morphology of electrophysiologically characterized and biocytin labeled cells. Cereb Cortex. 2004;14(6):690-701. doi: 10.1093/cercor/bhh029. PubMed PMID: 15054049.
20. Hay E, Schürmann F, Markram H, Segev I. Preserving axosomatic spiking features despite diverse dendritic morphology. J Neurophysiol. 2013;109(12):2972-81. doi: 10.1152/jn.00048.2013. PubMed PMID: 23536715.
