# Supplementary figures and images for: Selective recruitment of cortical neurons by electrical stimulation
Source: PLoS Comput Biol. 2019 Aug 26;15(8):e1007277. doi: 10.1371/journal.pcbi.1007277 (PMC6742409; doi:10.1371/journal.pcbi.1007277)

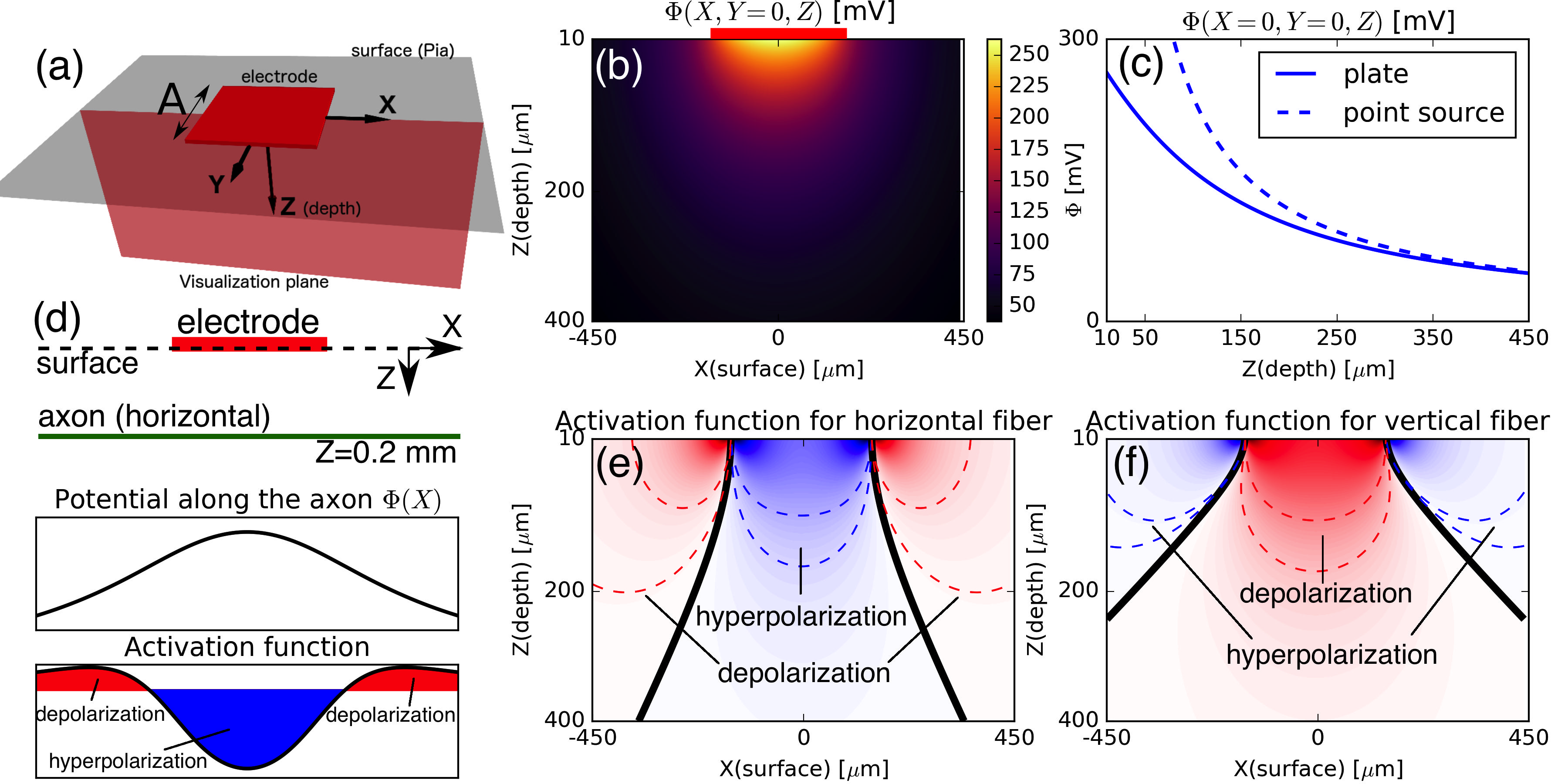

Supplement: S1 Fig — (a) Schematic representation of the electrode in the coordinate system (X,Y,Z). Electrode is located on the surface (gray), center of the coordinate system corresponds to the center of the electrode. (b) Electric potential Φ(X,Y,Z) on the plane Y = 0 (marked by red in panel (a)). (c) Comparison of the electric potential induced by point source (Eq (4), dashed curve) and finite-size square plate (Eq (3), solid curve) at varying depth Z and fixed X = Y = 0. (d) Top panel shows schematic representation of the electrode (red) and horizontally oriented axon (green) on (X,Z) plane (Y = 0). Bottom panels show potential Φ(x) and activating function ∂2Φ(x)/∂x2 along axonal fiber (anodal stimulation). (e,f) Activating function for horizontally (e) and vertically (f) oriented fibers as a function of coordinates X,Z on the plane Y = 0. Black solid curves separate areas of depolarization (red) and hyperpolarization (blue). Note that for cathodal stimulation the activating function is exactly opposite (area of depolarization and hyperpolarization are interchanged). (TIFF) [file pcbi.1007277.s002.tiff]
