# Supplementary material for: Selective recruitment of cortical neurons by electrical stimulation
Source: PLoS Comput Biol. 2019 Aug 26;15(8):e1007277. doi: 10.1371/journal.pcbi.1007277 (PMC6742409; doi:10.1371/journal.pcbi.1007277)
Supplement: S1 Table — (PDF) [file pcbi.1007277.s004.pdf]

S1 Table

Cell type	Size of dataset	References	Strain (Age)
Pyramidal cells (II/III)	26	[5, 18]	Wistar (P20-25), Sprague Dawley (25-36)
Pyramidal cells (IV)	33	[5, 19]	Wistar (P19-21), Sprague Dawley (25-36)
Slender-tufted PYs (Va)	43	[5, 6]	Wistar (P20-21), Sprague Dawley (25-36)
Thick-tufted PYs (Vb)	30	[20]	Wistar (P14)
Spiny stellate cells (IV)	9	[19]	Wistar (P19-21)
Layer I interneurons	118	[16]	Wistar (P13-16)
Basket cells (II/III)	97	[8]	Wistar (P13-15)
Basket cells (IV)	83	[8]	Wistar (P13-15)
Basket cells (V)	58	[8]	Wistar (P13-15)
Martinotti cells (II/III)	13	[15]	Wistar (P13-16)
Martinotti cells (IV)	9	[15]	Wistar (P13-16)
Martinotti cells (V)	7	[15]	Wistar (P13-16)
Bi-tufted cells (II/III)	18	[8]	Wistar (P13-15)
Bi-tufted cells (IV)	6	[8]	Wistar (P13-15)
Bi-tufted cells (V)	11	[8]	Wistar (P13-15)

S1 Table. Summary of datasets with reconstructed cells.
