# Supplementary material for: Selective recruitment of cortical neurons by electrical stimulation
Source: PLoS Comput Biol. 2019 Aug 26;15(8):e1007277. doi: 10.1371/journal.pcbi.1007277 (PMC6742409; doi:10.1371/journal.pcbi.1007277)
Supplement: S2 Table — PY–pyramidal cell, SC–spiny stellate cell, MC–Martinotti cell, BC–basket cell. (PDF) [file pcbi.1007277.s005.pdf]

S2 Table

Parameter	Description	Value		
C_m (μF/cm²)	Membrane capacitance	1		
η	Standard deviation of spontaneous background activity	0.4		
V_{Na} (mV)	Na^+ reversal potential	50		
V_K (mV)	K^+ reversal potential	-100		
V_{Ca} (mV)	Ca^{2+} reversal potential	120		
V_{shp} (mV)		2.5		
V_{th} (mV)	Threshold potential	25		
k_d				
α_{Ca}		0.005		
τ_{Ca} (ms^{-1})	Ca^{2+} decay time constant	200		
α_s^{exc} (ms^{-1})	Excitatory synaptic activation time constant	0.6		
α_s^{inh} (ms^{-1})	Inhibitory synaptic activation time constant	0.5		
β_s^{exc} (ms^{-1})	Excitatory synaptic deactivation time constant	0.2		
β_s^{inh} (ms^{-1})	Inhibitory synaptic deactivation time constant	0.1		
V_{syn}^{exc} (mV)	Excitatory synaptic reversal potential	0		
V_{syn}^{inh} (mV)	Inhibitory synaptic reversal potential	-80		
**Cell-Type Specific Parameters**		**PYs & SCs**	**MCs**	**BCs**
g_{Na} (mS/cm²)	Na^+ channel conductance	30	60	60
g_K (mS/cm²)	K^+ channel conductance	2.5	10	10
g_L (mS/cm²)	Leak channel conductance	0.6	0.4	0.4
g_{Ca} (mS/cm²)	Ca^{2+} channel conductance	0.1	0.1	0
g_{ahp} (mS/cm²)	Slow Ca^{2+}-dependent (after-hyperpolarization) K^+ channel conductance	0.5	0.75	0
V_L (mV)	Leak channel reversal potential	-67	-65	-65

Table 2. Model parameters for network simulations. PY – pyramidal cell, SC – spiny stellate cell, MC – Martinotti cell, BC – basket cell.
