# Supplementary material for: Selective recruitment of cortical neurons by electrical stimulation
Source: PLoS Comput Biol. 2019 Aug 26;15(8):e1007277. doi: 10.1371/journal.pcbi.1007277 (PMC6742409; doi:10.1371/journal.pcbi.1007277)
Supplement: S3 Table — PY–pyramidal neurons, BC–basket cells, SC–excitatory spiny stellate cells, MC–Martinotti cells. (PDF) [file pcbi.1007277.s006.pdf]

S3 Table

Type of cortical cells	Number of cells
PY II/III	200
BC II/III	50
PY+SC IV	200
BC IV	25
MC IV	25
PY Va (slender)	50
BC V	25
MC V	25

Table 3. Structure of the network. PY – pyramidal neurons, BC – basket cells, SC – excitatory spiny stellate cells, MC – Martinotti cells.
