# Supplementary material for: Selective recruitment of cortical neurons by electrical stimulation
Source: PLoS Comput Biol. 2019 Aug 26;15(8):e1007277. doi: 10.1371/journal.pcbi.1007277 (PMC6742409; doi:10.1371/journal.pcbi.1007277)
Supplement: S4 Table — PY–pyramidal neurons, BC–basket cells, SC–excitatory spiny stellate cells, MC–Martinotti cells. (PDF) [file pcbi.1007277.s007.pdf]

S4 Table

To		From	Type	Strength	Probability
PY, BC II/III		PY II/III	AMPA	0.75	0.1
PY II/III		PY, SC IV	AMPA	1.5	0.05
PY II/III		PY Va (slender)	AMPA	0.75	0.05
PY, SC, BC, MC IV		PY, SC IV	AMPA	1.5	0.1
PY, SC IV		PY II/III	AMPA	0.25	0.05
PY Va (slender)		PY Va (slender)	AMPA	0.75	0.1
BC, MC		PY Va (slender)	AMPA	0.75	0.1
PY II/III		BC II/III	GABA	3	0.25
PY II/III		MC IV,V	GABA	0.5	0.05
PY, SC IV		BC IV	GABA	3	0.25
PY, SC IV		MC IV	GABA	3	0.25
PY, SC IV		MC V	GABA	2	0.2
PY Va (slender)		BC V	GABA	3	0.25
PY Va (slender)		MC IV,V	GABA	0.5	0.05

Table 4. Connectivity within the network. PY – pyramidal neurons, BC – basket cells, SC – excitatory spiny stellate cells, MC – Martinotti cells.
